# Supplementary figures and images for: Dynamic genome plasticity during unisexual reproduction in the human fungal pathogen Cryptococcus deneoformans
Source: PLoS Genet. 2021 Nov 29;17(11):e1009935. doi: 10.1371/journal.pgen.1009935 (PMC8670703; doi:10.1371/journal.pgen.1009935)

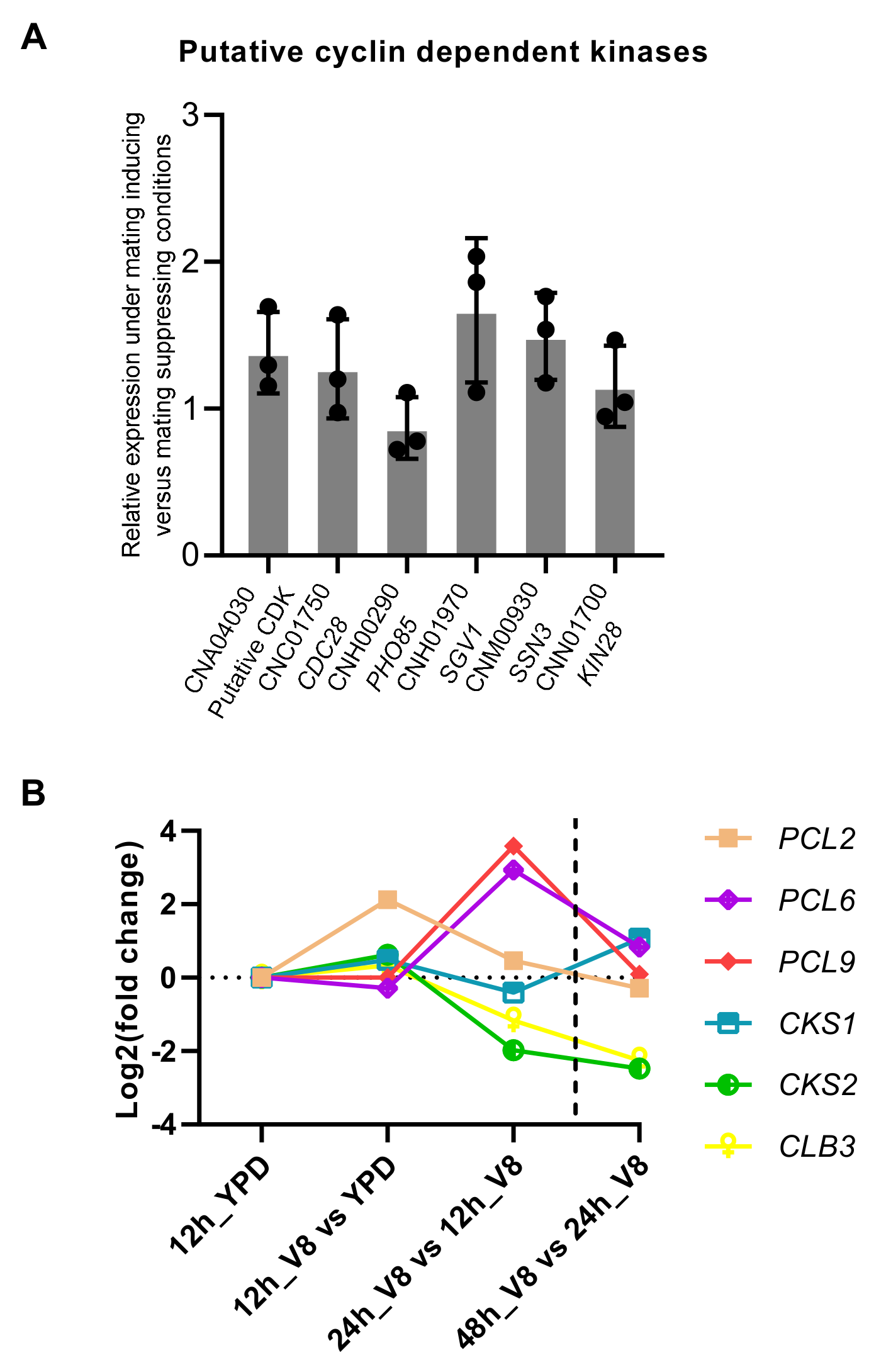

Supplement: S1 Fig — (A) Differential expression patterns of genes encoding putative cyclin dependent kinases in wild-type XL280α cells incubated for 36 hours on mating-inducing V8 agar medium versus nutrient-rich YPD agar medium were examined by qRT-PCR. (B) Relative expression levels for the six differentially expressed cell cycle regulators were extrapolated from a time-course transcriptional profiling study of the wild-type strain XL280α during unisexual reproduction [39]. Expression levels on YPD medium after incubation for 12h and V8 agar medium after incubation for 12h, 24h, and 48h were plotted for these putative cell cycle genes. A black dashed line was drawn to indicate the time point assayed for these genes in this study. (TIF) [file pgen.1009935.s001.tif]

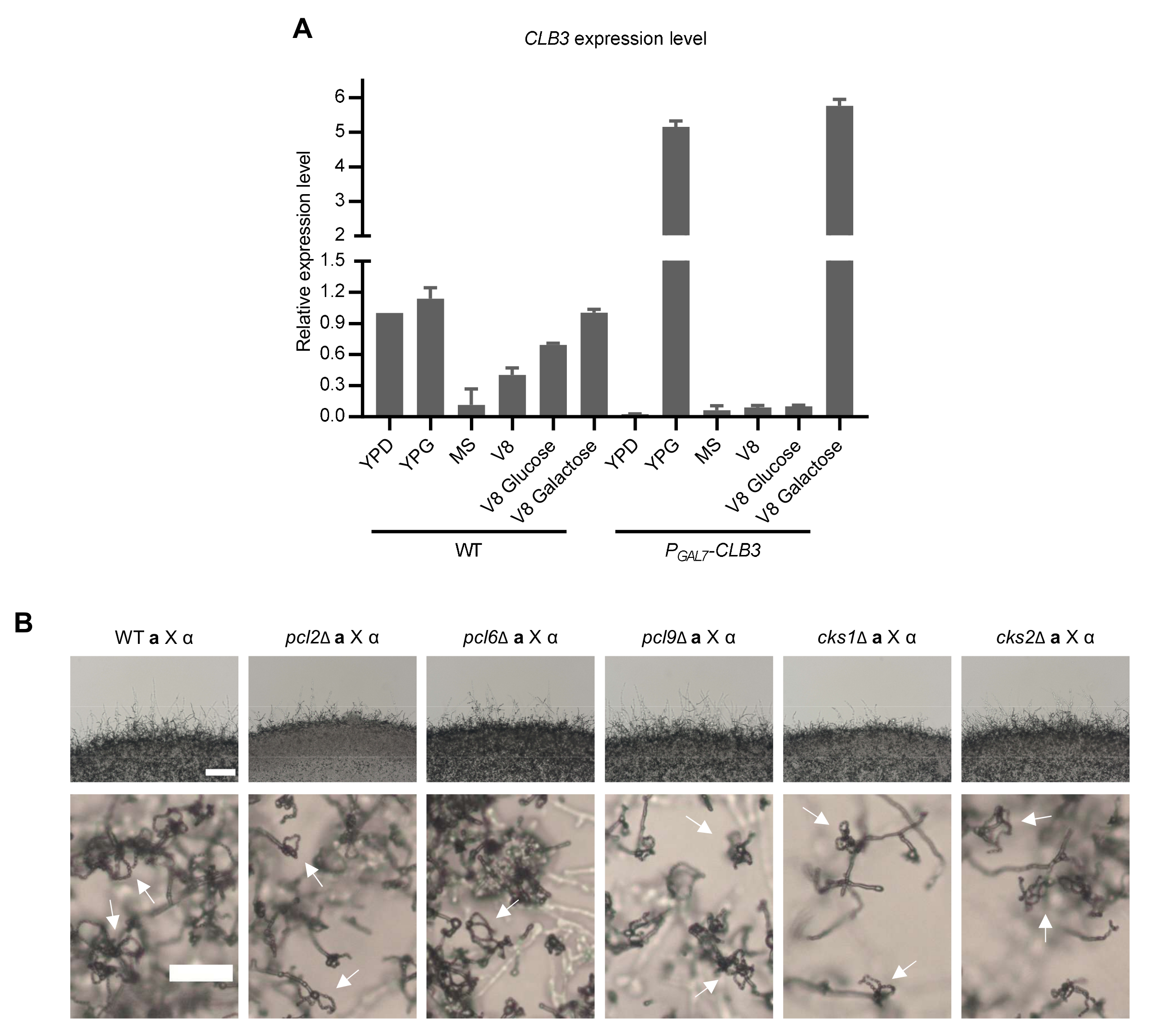

Supplement: S2 Fig — (A) CLB3 was expressed under the control of galactose-inducible promoter PGAL7. Compared to the expression level of the wild type on YPD agar medium, PGAL7-CLB3 was upregulated 5.2- and 5.8-fold on YPG and V8 galactose agar media and downregulated 38.8-, 15.4-, 10.9-, and 9.9-fold on YPD, MS, V8, and V8 glucose agar media, respectively. The error bars represent the standard deviation of the mean for three biological replicates. (B) MATa and MATα cells of wild type XL280 and deletion mutants for PCL2, PCL6, PCL9, CKS1, and CKS2 were equally mixed and inoculated on MS medium to assess bisexual hyphal growth and spore formation. Hyphal growth on the edge of each colony was imaged after three days and the scale bar represents 200 μm. Spore formation was imaged after eleven days and the scale bar represents 50 μm. (TIF) [file pgen.1009935.s002.tif]

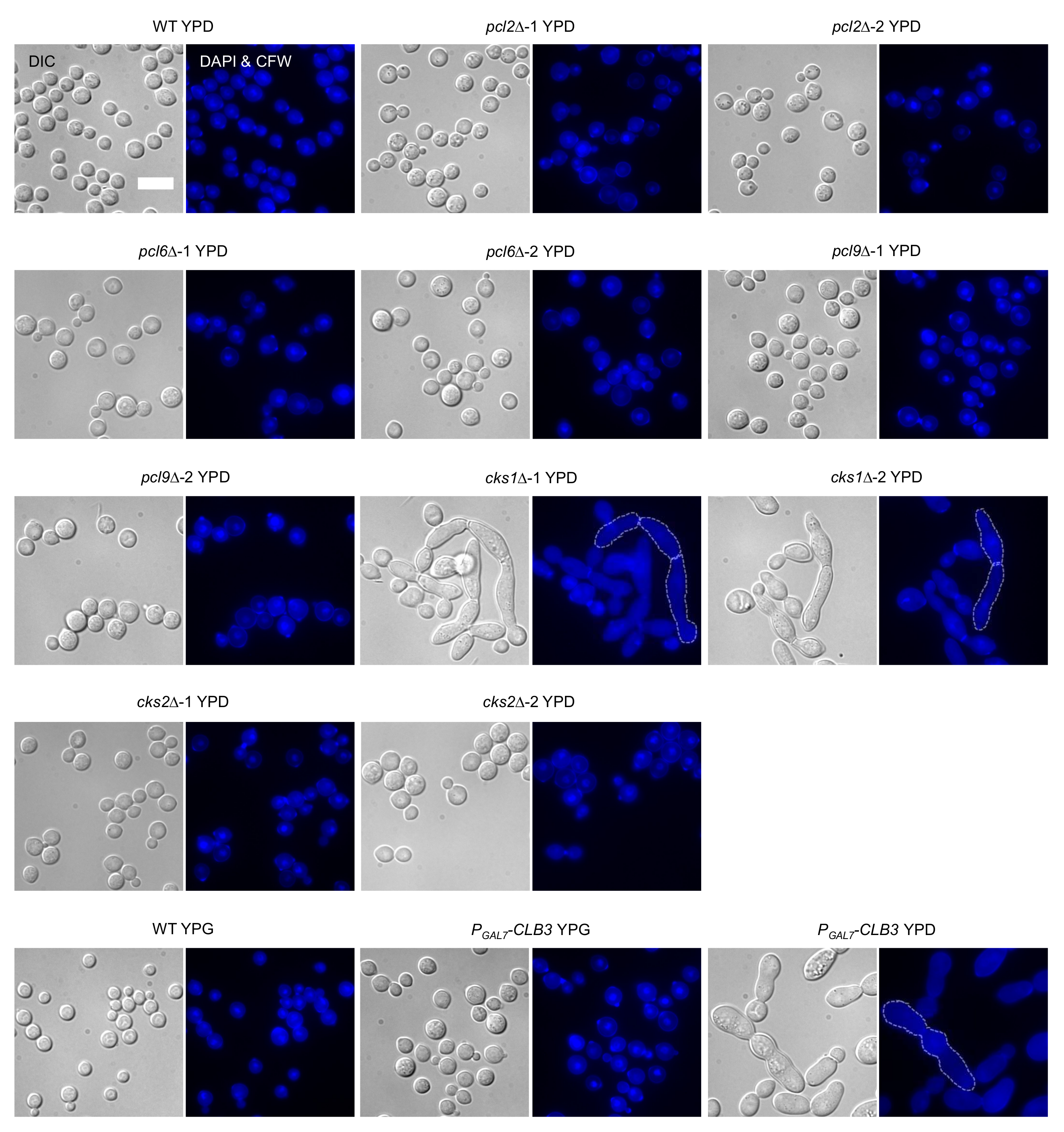

Supplement: S3 Fig — Wild type, deletion mutants of PCL2, PCL6, PCL9, CKS1, and CKS2 were grown in liquid YPD overnight, and the conditional expression strain for CLB3 was grown in liquid YPD and YPG medium overnight. Cells were stained with Calcofluor white and DAPI. The scale bar represents 10 μm. (TIF) [file pgen.1009935.s003.tif]

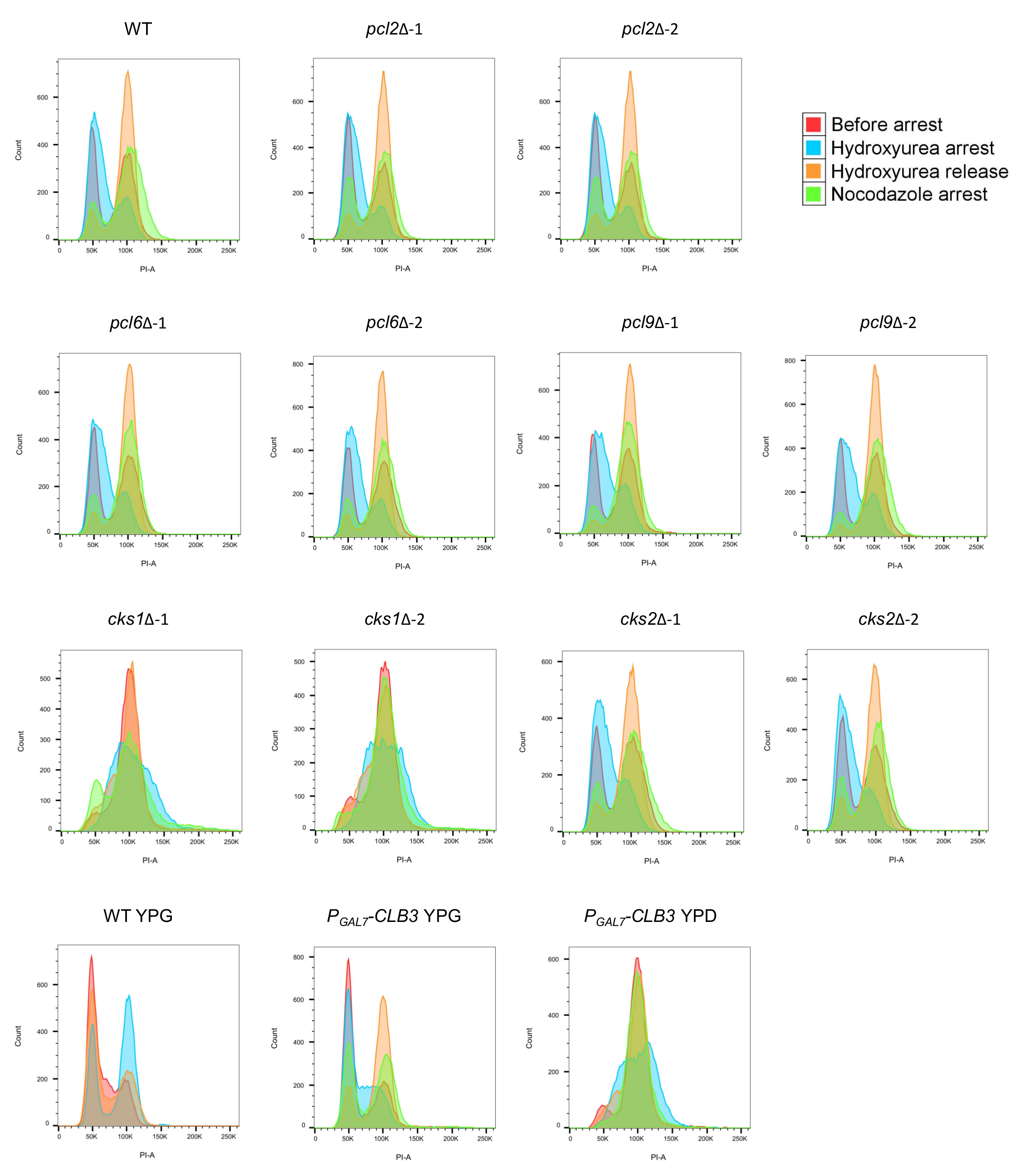

Supplement: S4 Fig — Overnight culture in YPD for the wild type and deletion mutant strains of PCL2, PCL6, PCL9, CKS1, and CKS2, and overnight culture in YPD for the conditional expression strain for CLB3 were arrested by hydroxyurea and nocodazole to assess whether these genes regulate cell cycle progression. Cells were arrested in G1 by hydroxyurea and released to S/G2 after removal of hydroxyurea. Nocodazole arrested cells at S/G2 phase. Ploidy for the cell populations were determined by FACS. (TIF) [file pgen.1009935.s004.tif]

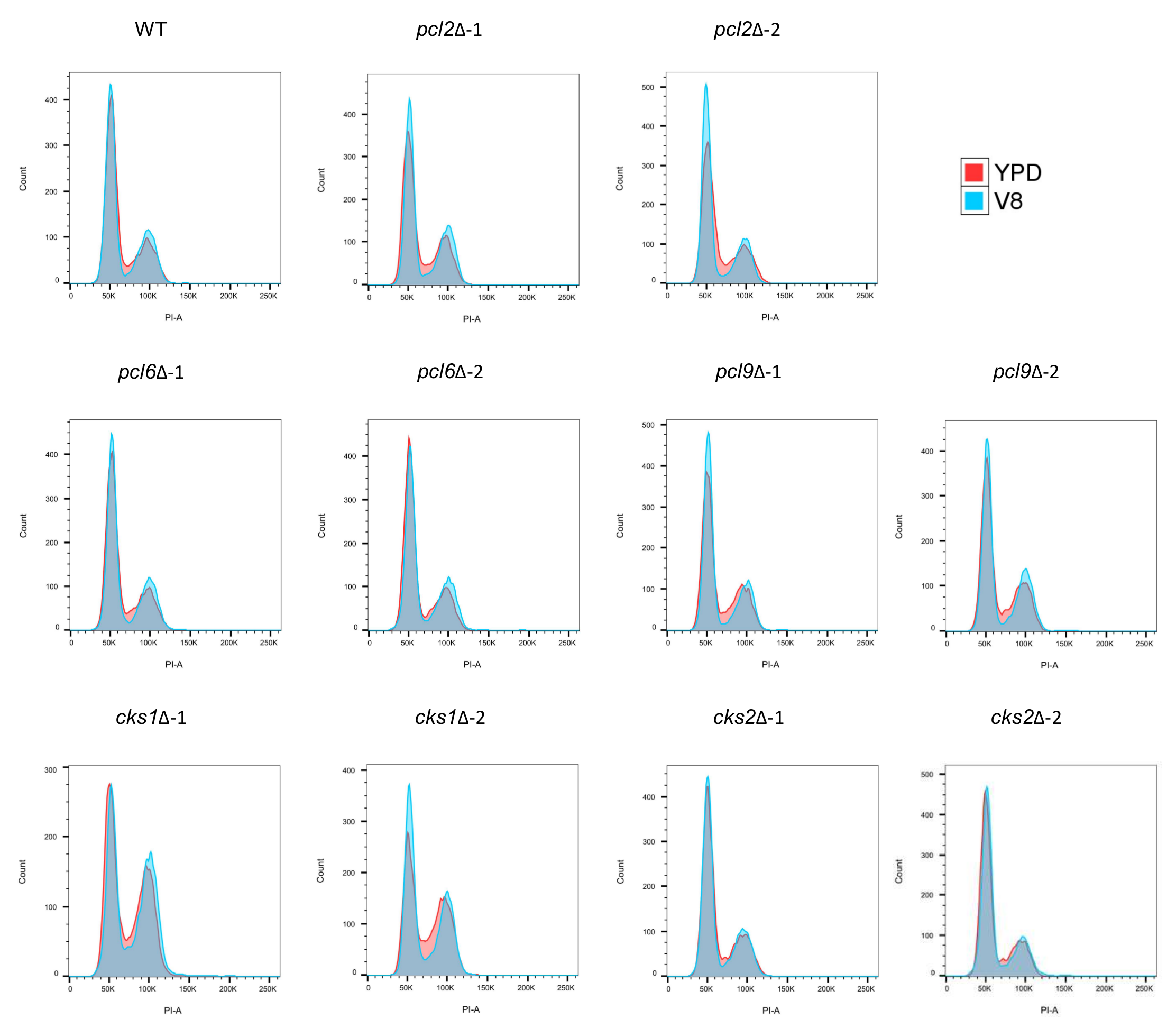

Supplement: S5 Fig — Wild type and deletion mutants of PCL2, PCL6, PCL9, CKS1, and CKS2 were grown on YPD and V8 agar media for 24 hours. (TIF) [file pgen.1009935.s005.tif]

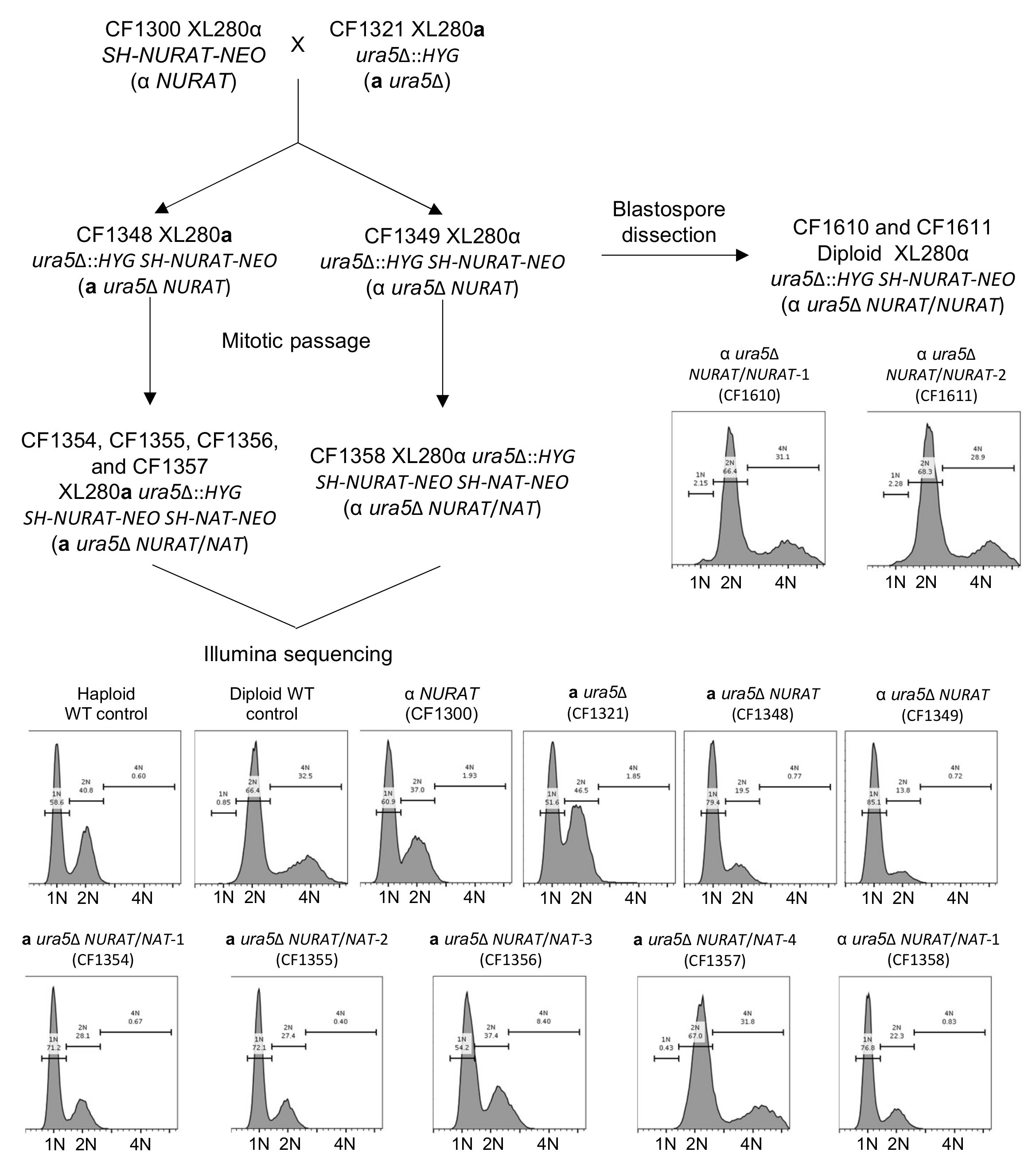

Supplement: S6 Fig — A MATα NURAT strain (CF1300 XL280α SH-NURAT-NEO) was crossed with a MATa ura5Δ strain (CF1321 XL280a ura5Δ::HYG) to generate MATa and MATα ura5Δ NURAT strains (CF1348 and CF1349). The MATa ura5Δ NURAT/NAT-1, -2, -3, and -4 strains (CF1354, CF1355, CF1356, and CF1347) and the MATα ura5Δ NURAT/NAT-1 strain (CF1358) were generated through mitotic passages of CF1348 and CF1349, respectively. All above nine strains were subjected to Illumina whole-genome sequencing. Diploid MATα ura5Δ NURAT/NURAT strains (CF1610 and CF1611 α/α ura5Δ/ura5Δ NURAT/NURAT) were generated by dissecting blastospores from CF1349. Ploidy of all strains were confirmed by FACS. (TIF) [file pgen.1009935.s006.tif]

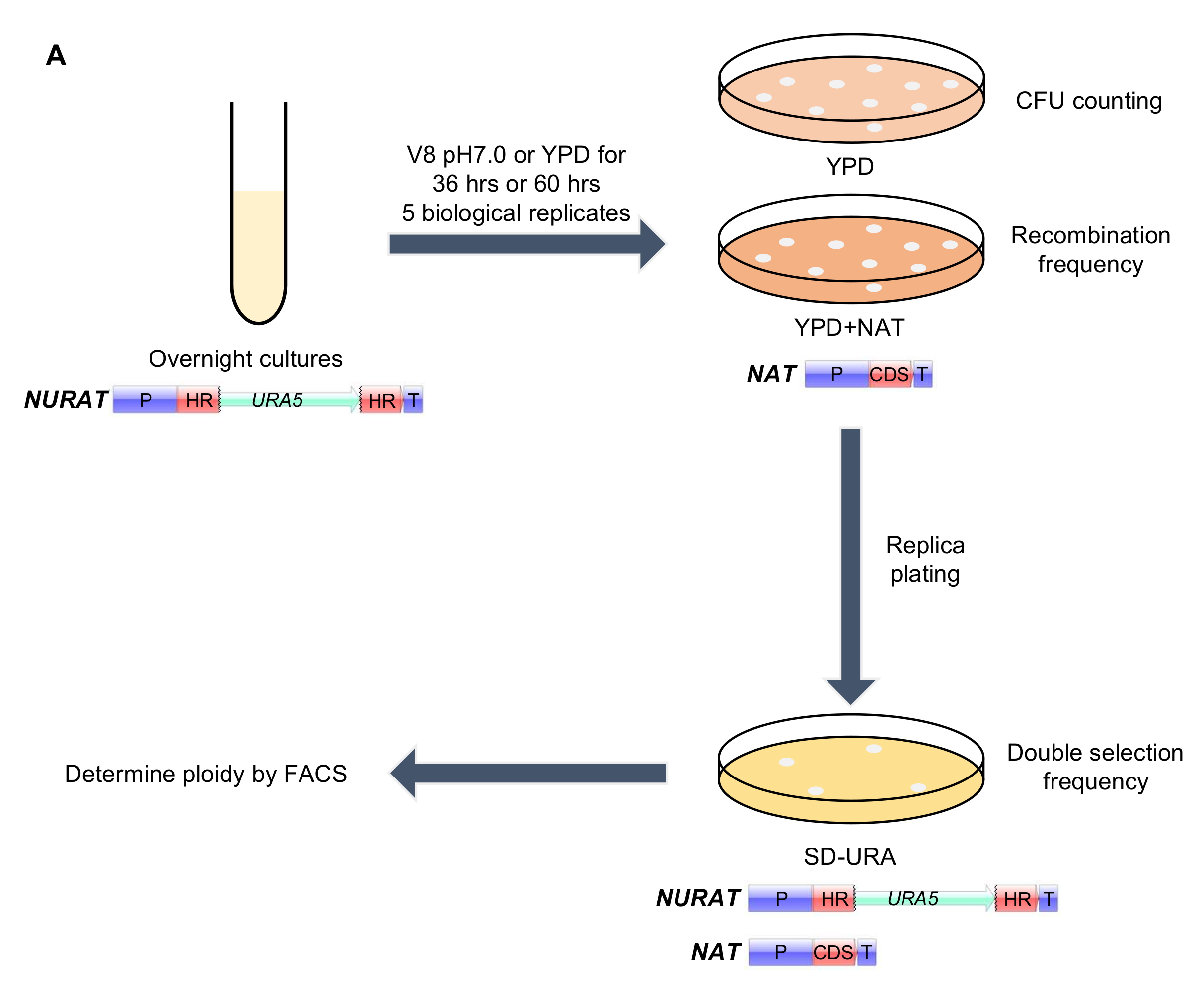

Supplement: S7 Fig — Overnight cultures of strains carrying the NURAT construct were washed and inoculated on V8 or YPD medium for the designated time period. Cells were then plated on YPD medium supplemented with nourseothricin to select for cells with a recombined, functional NAT construct. Colonies derived from these cells were replica plated onto SD-URA medium to screen for NATR cells that retained an intact NURAT construct. NATR, Ura+ colonies were then tested for ploidy by FACS. (TIF) [file pgen.1009935.s007.tif]

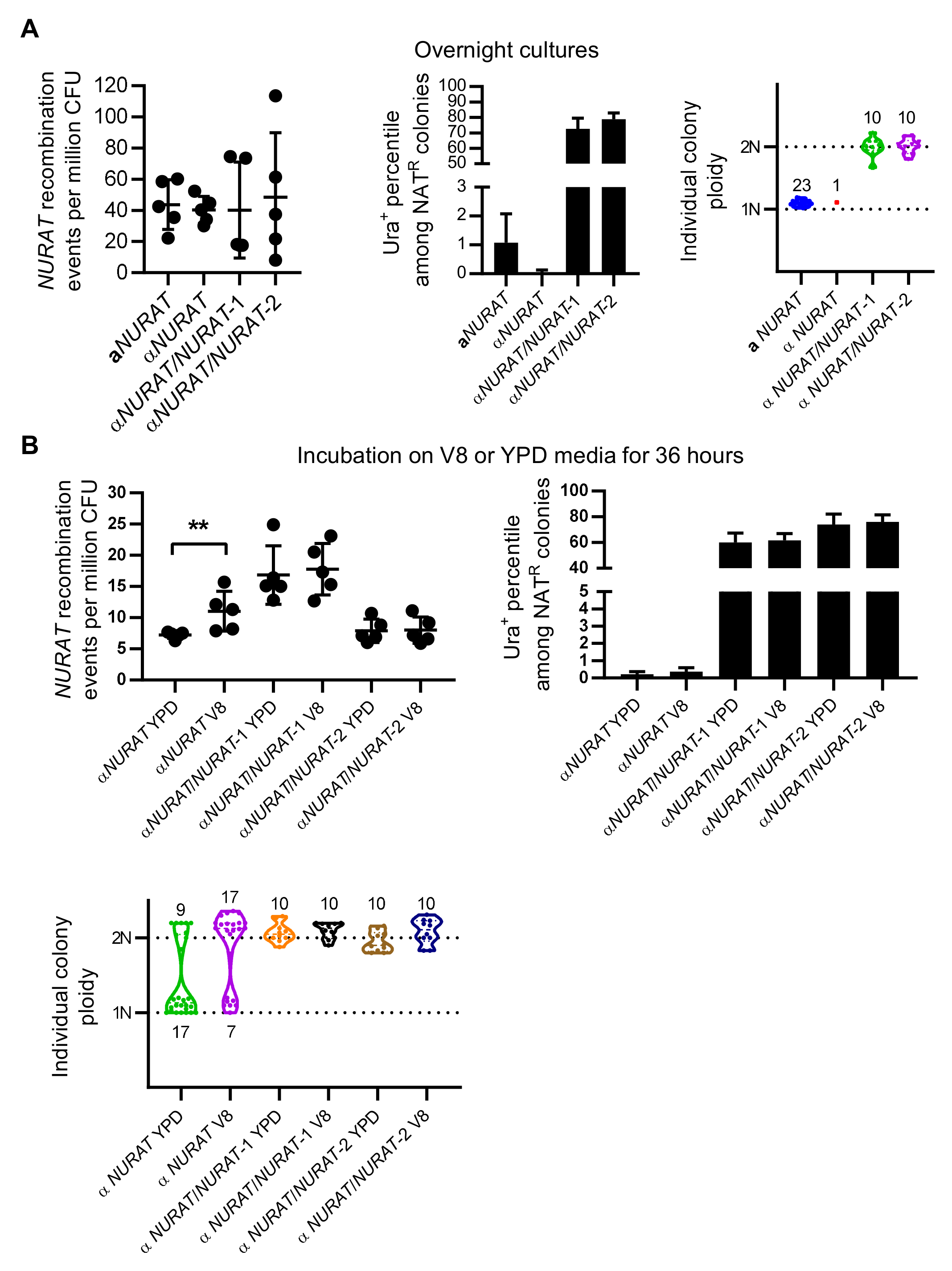

Supplement: S8 Fig — Frequencies of NURAT recombination in haploid strains MATa NURAT (CF1348, only overnight culture was tested) and MATα NURAT (CF1349), and diploid strains MAT α NURAT/NURAT-1 and MATα NURAT/NURAT-2 (CF1610 and CF1611) grown (A) as overnight cultures in liquid YPD medium and (B) on V8 or YPD agar medium for 36 hours (scatter dot plots). NATR colonies were replica-plated onto SD-URA medium to obtain NATR, Ura+ colonies (bar graphs), and ploidy for these colonies was assessed by FACS (violin plots). Mean values of five independent experiments were plotted for the NURAT recombination frequencies and the Ura+ percentiles among NATR colonies; error bars represent standard deviations. Student’s T-test was performed for each pairwise comparison. ** indicates 0.001 < p ≤ 0.01. (TIF) [file pgen.1009935.s008.tif]

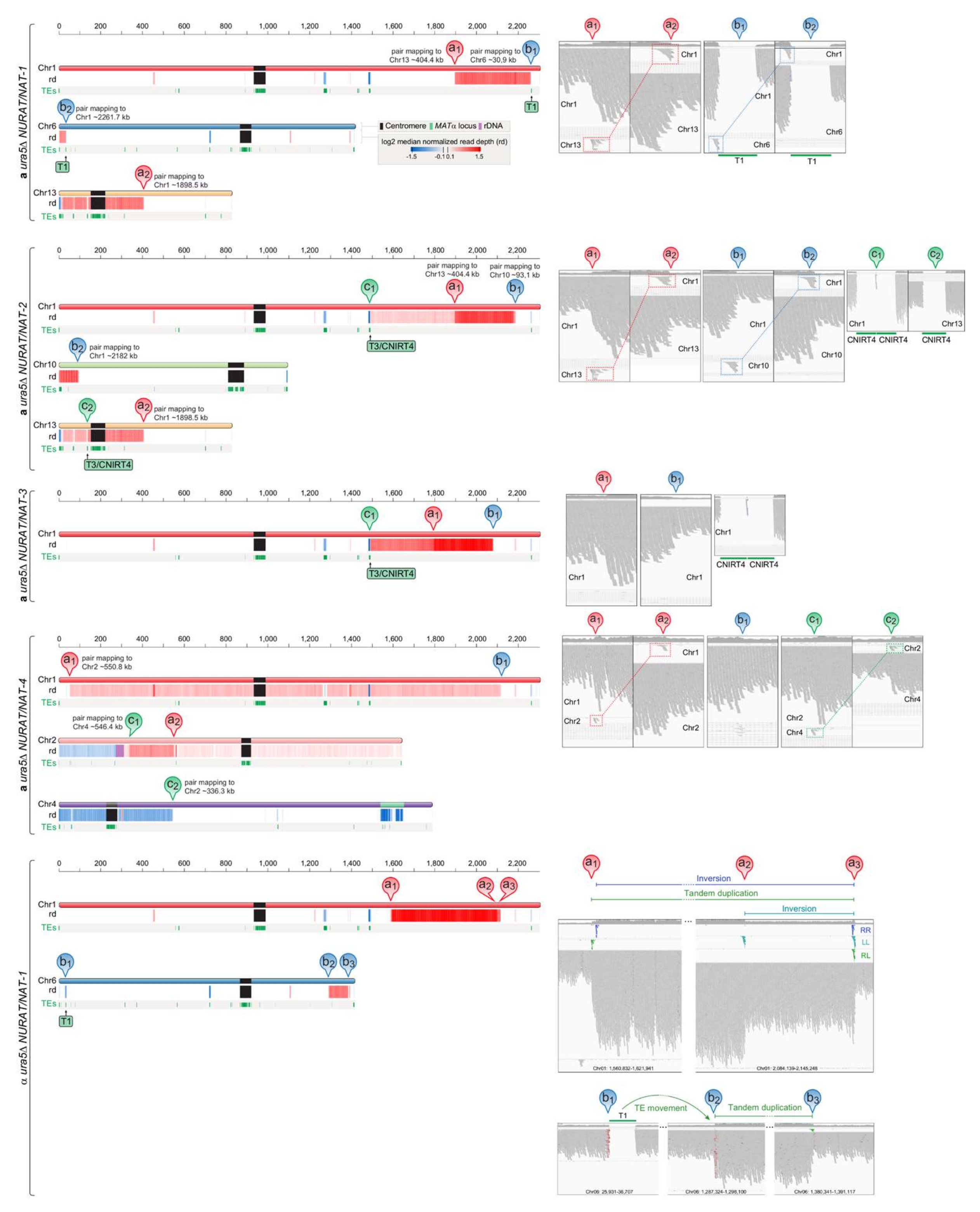

Supplement: S9 Fig — For each NURAT/NAT progeny, sequencing reads at the borders of segmentally duplicated regions were analyzed. Blue, red, and green bubbles indicate forward and reverse reads that were aligned to two different chromosomal positions. Sequence alignments of these reads were shown in the panels on the right of the chromosome diagrams. Chimeric reads aligning to two different chromosomal positions were highlighted in connected boxes. Sequencing reads aligned to segmentally duplicated regions from three chromosomes were identified in the MATa ura5Δ NURAT/NAT-1, -2, and -4 strains, suggesting fusion of these regions. T1 and T3/CNIRT4 transposable element movements were detected flanking some of the regions in the MATa ura5Δ NURAT/NAT-1, -2, and -3 strains and the MATα ura5Δ NURAT/NAT-1 strain. In the MATα ura5Δ NURAT/NAT-1 strain, tandem duplication and inversion events were detected in the segmentally duplicated regions. (TIF) [file pgen.1009935.s009.tif]

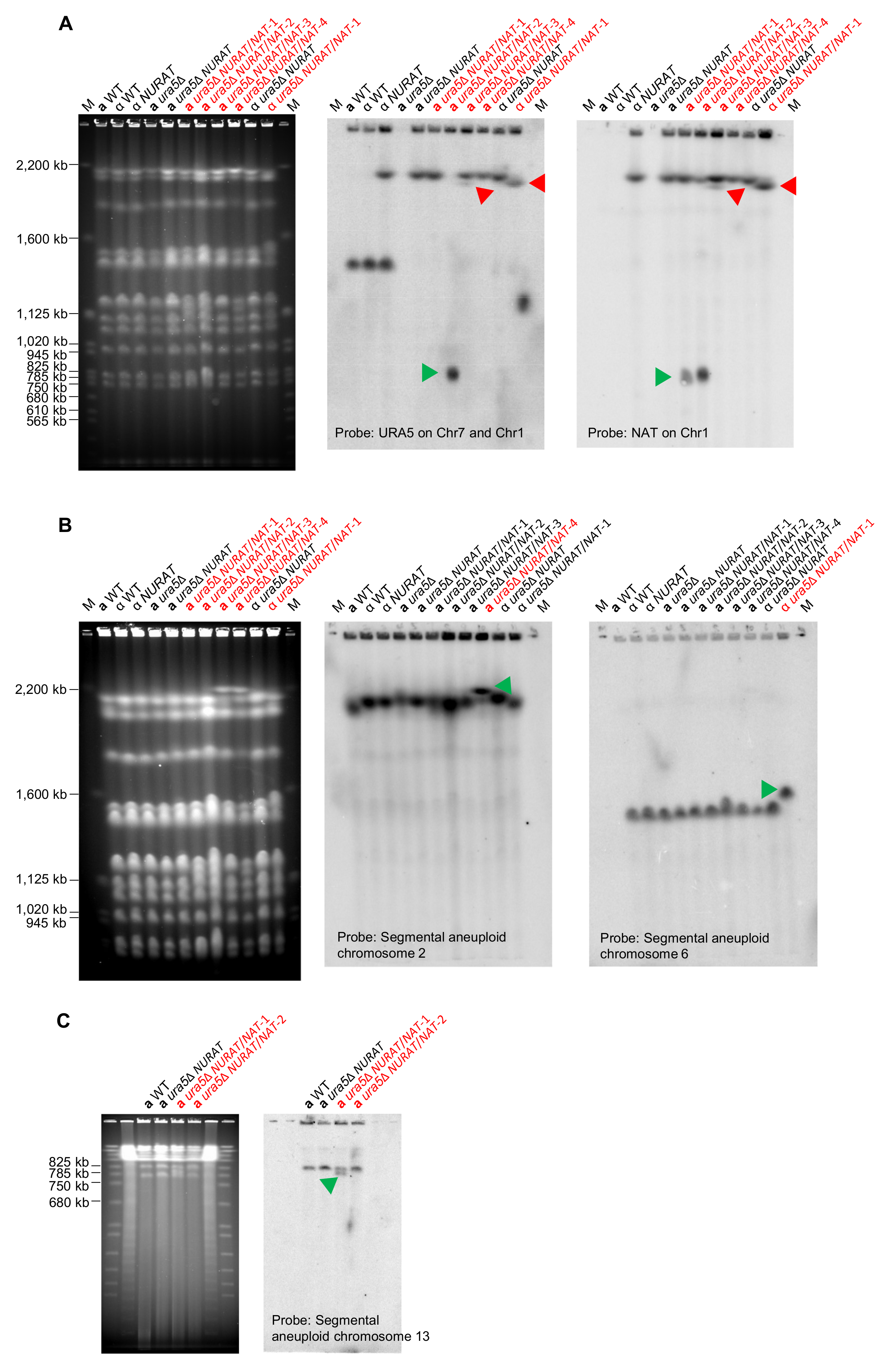

Supplement: S10 Fig — CHEF gel electrophoresis separation of chromosomes was performed under different conditions to separate larger or smaller chromosomes. Karyotypic changes (highlighted in green and red arrows) were observed for strains with segmental aneuploidy (MATa ura5Δ NAT/NURAT-1, -2, -3, -4 and MATα ura5Δ NAT/NURAT-1) compared with wild type and parental strains. Chromoblot analyses with probes recognizing (A) URA5 and NAT, and segmental aneuploid portions of (B) Chrs 2 and 6 and (C) Chr 13 confirmed the karyotypic changes. Strains are highlighted in red when the probed sequences are within segmental aneuploid regions. (TIF) [file pgen.1009935.s010.tif]

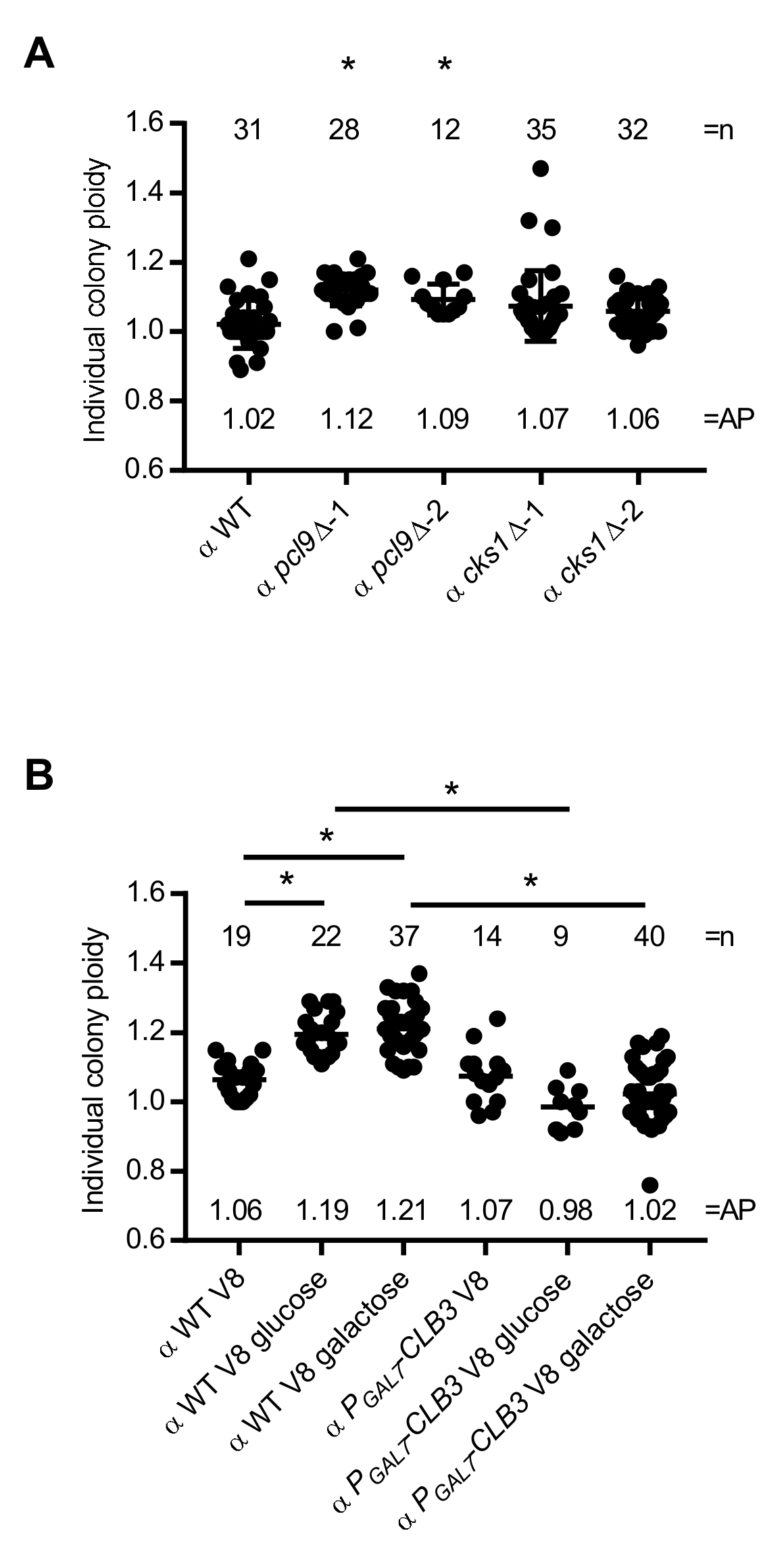

Supplement: S11 Fig — Individual colonies with ploidies above 1.6 were identified as outliers and removed from Fig 5 and data were replotted for (A) pcl9Δ and cks1Δ mutants and (B) PGAL7-CLB3. Student’s t-tests with Bonferroni correction for 4 and 9 repeated tests were performed for each pairwise comparison for panel A and panel B, respectively. p value lower than 0.0125 (A) or 0.0056 (B) was considered statistically significant (*). (TIF) [file pgen.1009935.s011.tif]
