# Supplementary material for: Dynamic genome plasticity during unisexual reproduction in the human fungal pathogen Cryptococcus deneoformans
Source: PLoS Genet. 2021 Nov 29;17(11):e1009935. doi: 10.1371/journal.pgen.1009935 (PMC8670703; doi:10.1371/journal.pgen.1009935)
Supplement: S3 Table — (DOCX) [file pgen.1009935.s014.docx]

**S3 Table. Mitotically passaged yeast cells maintained stable ploidy.**

| Strains | Blastospore ploidy | Single colony tested | Diploid (D) | Haploid (H) | Mixed H/D |
| --- | --- | --- | --- | --- | --- |
| XL280α | Diploid-1 | 8 | 8 | 0 | 0 |
|  | Diploid-2 | 8 | 8 | 0 | 0 |
|  | Diploid-3 | 8 | 8 | 0 | 0 |
|  | Diploid-4 | 8 | 8 | 0 | 0 |
| *pcl6*∆-1 | Diploid-1 | 8 | 8 | 0 | 0 |
|  | Diploid-2 | 8 | 8 | 0 | 0 |
|  | Diploid-3 | 8 | 8 | 0 | 0 |
|  | Diploid-4 | 8 | 8 | 0 | 0 |
|  | Mixed H/D-1 | 10 | 0 | 10 | 0 |
|  | Mixed H/D-2 | 10 | 3 | 6 | 1 |
|  | Mixed H/D-3 | 10 | 5 | 5 | 0 |
| *pcl6*∆-2 | Diploid-1 | 8 | 8 | 0 | 0 |
|  | Diploid-2 | 8 | 8 | 0 | 0 |
|  | Diploid-3 | 8 | 8 | 0 | 0 |
|  | Diploid-4 | 8 | 8 | 0 | 0 |
|  | Mixed H/D-1 | 10 | 1 | 9 | 0 |
|  | Mixed H/D-2 | 10 | 5 | 5 | 0 |
|  | Mixed H/D-3 | 10 | 3 | 7 | 0 |
