# Supplementary material for: Dynamic genome plasticity during unisexual reproduction in the human fungal pathogen Cryptococcus deneoformans
Source: PLoS Genet. 2021 Nov 29;17(11):e1009935. doi: 10.1371/journal.pgen.1009935 (PMC8670703; doi:10.1371/journal.pgen.1009935)
Supplement: S5 Table — (DOCX) [file pgen.1009935.s016.docx]

**S5 Table. Strains and plasmids used in this study.**

| **Strain name** | **Genotype** | **Ploidy** | **Sources** |
| --- | --- | --- | --- |
| XL280α | WT | Haploid | [1] |
| XL280**a** | Congenic strain of XL280α | Haploid | [2] |
| MN142.6 | *MAT*α/α *ura5*∆::*NAT*/*ura5*∆::*NEO* | Diploid | [3] |
| CF1399 | *MAT*α *pcl2*∆::*NEO*-1 | Haploid | This study |
| CF1535 | *MAT*α *pcl2*∆::*NEO*-2 | Haploid | This study |
| CF1360 | *MAT*α *pcl6*∆::*NEO*-1 | Haploid | This study |
| CF1361 | *MAT*α *pcl6*∆::*NEO*-2 | Haploid | This study |
| CF1765 | *MAT*α *pcl9*∆::*NEO*-1 | Haploid | This study |
| CF1768 | *MAT*α *pcl9*∆::*NEO*-2 | Haploid | This study |
| CF1367 | *MAT*α *cks1*∆::*NEO*-1 | Haploid | This study |
| CF1414 | *MAT*α *cks1*∆::*NEO*-2 | Haploid | This study |
| CF1379 | *MAT*α *cks2*∆::*NEO*-1 | Haploid | This study |
| CF1387 | *MAT*α *cks2*∆::*NEO*-2 | Haploid | This study |
| CF1715 | *MAT*α *P_GAL7_-CLB3-NEO* | Haploid | This study |
| CF1300 | *MAT*α *SH-NURAT-NEO* | Haploid | This study |
| CF1321 | *MAT***a** *ura5*∆::*HYG* | Haploid | This study |
| CF1348 | *MAT***a** *ura5*∆::*HYG SH-NURAT-NEO* | Haploid | This study |
| CF1349 | *MAT*α *ura5*∆::*HYG SH-NURAT-NEO* | Haploid | This study |
| CF1610 | *MAT*α/α *ura5*∆::*HYG*/*ura5*∆::*HYG SH-NURAT-NEO*/*SH-NURAT-NEO*-1 | Diploid | This study |
| CF1611 | *MAT*α/α *ura5*∆::*HYG*/*ura5*∆::*HYG SH-NURAT-NEO*/*SH-NURAT-NEO*-2 | Diploid | This study |
| CF1354 | *MAT***a** *ura5*∆::*HYG SH-NURAT-NEO*/*SH-NAT-NEO*-1 | Haploid | This study |
| CF1355 | *MAT***a** *ura5*∆::*HYG SH-NURAT-NEO*/*SH-NAT-NEO*-2 | Haploid | This study |
| CF1356 | *MAT***a** *ura5*∆::*HYG SH-NURAT-NEO*/*SH-NAT-NEO*-3 | Haploid | This study |
| CF1357 | *MAT***a**/**a** *ura5*∆::*HYG*/*ura5*∆::*HYG SH-NURAT-NEO*/*SH-NAT-NEO*-4 | Diploid | This study |
| CF1358 | *MAT*α *ura5*∆::*HYG SH-NURAT-NEO*/*SH-NAT-NEO*-1 | Haploid | This study |
| CF1510 | *MAT***a** *pcl2*∆::*NEO* | Haploid | This study |
| CF1534 | *MAT***a** *pcl6*∆::*NEO* | Haploid | This study |
| CF1798 | *MAT***a** *pcl9*∆::*NEO* | Haploid | This study |
| CF1526 | *MAT***a** *cks1*∆::*NEO* | Haploid | This study |
| CF1516 | *MAT***a** *cks2*∆::*NEO* | Haploid | This study |
| CF1779 | *MAT*α *pcl2*∆::*NEO ura5*∆::*HYG SH-NURAT-NEO*-1 | Haploid | This study |
| CF1780 | *MAT*α *pcl2*∆::*NEO ura5*∆::*HYG SH-NURAT-NEO*-2 | Haploid | This study |
| CF1773 | *MAT*α *pcl6*∆::*NEO ura5*∆::*HYG SH-NURAT-NEO* | Haploid | This study |
| CF1774 | *MAT***a** *pcl6*∆::*NEO ura5*∆::*HYG SH-NURAT-NEO* | Haploid | This study |
| CF1806 | *MAT*α *pcl9*∆::*NEO ura5*∆::*HYG SH-NURAT-NEO*-1 | Haploid | This study |
| CF1807 | *MAT*α *pcl9*∆::*NEO ura5*∆::*HYG SH-NURAT-NEO*-2 | Haploid | This study |
| CF1784 | *MAT*α *cks1*∆::*NEO ura5*∆::*HYG SH-NURAT-NEO*-1 | Haploid | This study |
| CF1787 | *MAT*α *cks1*∆::*NEO ura5*∆::*HYG SH-NURAT-NEO*-2 | Haploid | This study |
| CF1770 | *MAT*α *cks2*∆::*NEO ura5*∆::*HYG SH-NURAT-NEO*-1 | Haploid | This study |
| CF1772 | *MAT*α *cks2*∆::*NEO ura5*∆::*HYG SH-NURAT-NEO*-2 | Haploid | This study |
| CF1835 | *MAT*α *P_GAL7_-CLB3-NEO ura5*∆::*HYG SH-NURAT-NEO*-1 | Haploid | This study |
| **Genotype** | **Blastospores dissected in this study*** | **Ploidy**** | |
| WT | XL280α blastospore 1 (47/49, 10 sites, site 1 5/5) | Diploid | |
|  | XL280α blastospore 2 (47/49, 10 sites, site 1 5/5) | Diploid | |
|  | XL280α blastospore 3 (47/49, 10 sites, site 1 5/5) |  | |
|  | XL280α blastospore 4 (47/49, 10 sites, site 1 5/5) |  | |
|  | XL280α blastospore 5 (47/49, 10 sites, site 1 5/5) |  | |
|  | XL280α blastospore 6 (47/49, 10 sites, site 2 6/6) | Diploid | |
|  | XL280α blastospore 7 (47/49, 10 sites, site 2 6/6) | Diploid | |
|  | XL280α blastospore 8 (47/49, 10 sites, site 2 6/6) |  | |
|  | XL280α blastospore 9 (47/49, 10 sites, site 2 6/6) |  | |
|  | XL280α blastospore 10 (47/49, 10 sites, site 2 6/6) |  | |
|  | XL280α blastospore 11 (47/49, 10 sites, site 2 6/6) |  | |
|  | XL280α blastospore 12 (47/49, 10 sites, site 3 7/7) | Diploid | |
|  | XL280α blastospore 13 (47/49, 10 sites, site 3 7/7) | Diploid | |
|  | XL280α blastospore 14 (47/49, 10 sites, site 3 7/7) |  | |
|  | XL280α blastospore 15 (47/49, 10 sites, site 3 7/7) |  | |
|  | XL280α blastospore 16 (47/49, 10 sites, site 3 7/7) |  | |
|  | XL280α blastospore 17 (47/49, 10 sites, site 3 7/7) |  | |
|  | XL280α blastospore 18 (47/49, 10 sites, site 3 7/7) |  | |
|  | XL280α blastospore 19 (47/49, 10 sites, site 4 3/3) | Diploid | |
|  | XL280α blastospore 20 (47/49, 10 sites, site 4 3/3) | Diploid | |
|  | XL280α blastospore 21 (47/49, 10 sites, site 4 3/3) |  | |
|  | XL280α blastospore 22 (47/49, 10 sites, site 5 6/6) | Aneuploid | |
|  | XL280α blastospore 23 (47/49, 10 sites, site 5 6/6) | Aneuploid | |
|  | XL280α blastospore 24 (47/49, 10 sites, site 5 6/6) |  | |
|  | XL280α blastospore 25 (47/49, 10 sites, site 5 6/6) |  | |
|  | XL280α blastospore 26 (47/49, 10 sites, site 5 6/6) |  | |
|  | XL280α blastospore 27 (47/49, 10 sites, site 5 6/6) |  | |
|  | XL280α blastospore 28 (47/49, 10 sites, site 6 5/5) | Diploid | |
|  | XL280α blastospore 29 (47/49, 10 sites, site 6 5/5) | Diploid | |
|  | XL280α blastospore 30 (47/49, 10 sites, site 6 5/5) |  | |
|  | XL280α blastospore 31 (47/49, 10 sites, site 6 5/5) |  | |
|  | XL280α blastospore 32 (47/49, 10 sites, site 6 5/5) |  | |
|  | XL280α blastospore 33 (47/49, 10 sites, site 7 4/4) | Diploid | |
|  | XL280α blastospore 34 (47/49, 10 sites, site 7 4/4) | Diploid | |
|  | XL280α blastospore 35 (47/49, 10 sites, site 7 4/4) |  | |
|  | XL280α blastospore 36 (47/49, 10 sites, site 7 4/4) |  | |
|  | XL280α blastospore 37 (47/49, 10 sites, site 8 3/4) | Diploid | |
|  | XL280α blastospore 38 (47/49, 10 sites, site 8 3/4) | Diploid | |
|  | XL280α blastospore 39 (47/49, 10 sites, site 8 3/4) |  | |
|  | XL280α blastospore 40 (47/49, 10 sites, site 9 5/5) | Diploid | |
|  | XL280α blastospore 41 (47/49, 10 sites, site 9 5/5) | Diploid | |
|  | XL280α blastospore 42 (47/49, 10 sites, site 9 5/5) |  | |
|  | XL280α blastospore 43 (47/49, 10 sites, site 9 5/5) |  | |
|  | XL280α blastospore 44 (47/49, 10 sites, site 9 5/5) |  | |
|  | XL280α blastospore 45 (47/49, 10 sites, site 10 3/4) | Diploid | |
|  | XL280α blastospore 46 (47/49, 10 sites, site 10 3/4) | Diploid | |
|  | XL280α blastospore 47 (47/49, 10 sites, site 10 3/4) |  | |
| *pcl2*∆-1 | CF1399 blastospore 1 (41/49, 15 sites, site 1 3/3) | Diploid | |
|  | CF1399 blastospore 2 (41/49, 15 sites, site 1 3/3) | Diploid | |
|  | CF1399 blastospore 3 (41/49, 15 sites, site 1 3/3) |  | |
|  | CF1399 blastospore 4 (41/49, 15 sites, site 2 4/4) | Diploid | |
|  | CF1399 blastospore 5 (41/49, 15 sites, site 2 4/4) | Diploid | |
|  | CF1399 blastospore 6 (41/49, 15 sites, site 2 4/4) |  | |
|  | CF1399 blastospore 7 (41/49, 15 sites, site 2 4/4) |  | |
|  | CF1399 blastospore 8 (41/49, 15 sites, site 3 5/5) | Diploid | |
|  | CF1399 blastospore 9 (41/49, 15 sites, site 3 5/5) | Diploid | |
|  | CF1399 blastospore 10 (41/49, 15 sites, site 3 5/5) |  | |
|  | CF1399 blastospore 11 (41/49, 15 sites, site 3 5/5) |  | |
|  | CF1399 blastospore 12 (41/49, 15 sites, site 3 5/5) |  | |
|  | CF1399 blastospore 13 (41/49, 15 sites, site 4 5/5) | Diploid | |
|  | CF1399 blastospore 14 (41/49, 15 sites, site 4 5/5) | Diploid | |
|  | CF1399 blastospore 15 (41/49, 15 sites, site 4 5/5) |  | |
|  | CF1399 blastospore 16 (41/49, 15 sites, site 4 5/5) |  | |
|  | CF1399 blastospore 17 (41/49, 15 sites, site 4 5/5) |  | |
|  | CF1399 blastospore 18 (41/49, 15 sites, site 5 2/2) | Diploid | |
|  | CF1399 blastospore 19 (41/49, 15 sites, site 5 2/2) | Diploid | |
|  | CF1399 blastospore 20 (41/49, 15 sites, site 6 3/3) | Diploid | |
|  | CF1399 blastospore 21 (41/49, 15 sites, site 6 3/3) | Diploid | |
|  | CF1399 blastospore 22 (41/49, 15 sites, site 6 3/3) |  | |
|  | CF1399 blastospore 23 (41/49, 15 sites, site 7 1/3) | Diploid | |
|  | CF1399 blastospore 24 (41/49, 15 sites, site 9 3/3) | Diploid | |
|  | CF1399 blastospore 25 (41/49, 15 sites, site 9 3/3) | Diploid | |
|  | CF1399 blastospore 26 (41/49, 15 sites, site 9 3/3) |  | |
|  | CF1399 blastospore 27 (41/49, 15 sites, site 10 2/2) | Diploid | |
|  | CF1399 blastospore 28 (41/49, 15 sites, site 10 2/2) | Diploid | |
|  | CF1399 blastospore 29 (41/49, 15 sites, site 11 4/4) | Diploid | |
|  | CF1399 blastospore 30 (41/49, 15 sites, site 11 4/4) | Diploid | |
|  | CF1399 blastospore 31 (41/49, 15 sites, site 11 4/4) |  | |
|  | CF1399 blastospore 32 (41/49, 15 sites, site 11 4/4) |  | |
|  | CF1399 blastospore 33 (41/49, 15 sites, site 12 2/2) | Diploid | |
|  | CF1399 blastospore 34 (41/49, 15 sites, site 12 2/2) | Diploid | |
|  | CF1399 blastospore 35 (41/49, 15 sites, site 13 2/3) | Diploid | |
|  | CF1399 blastospore 36 (41/49, 15 sites, site 13 2/3) | Diploid | |
|  | CF1399 blastospore 37 (41/49, 15 sites, site 14 2/4) | Diploid | |
|  | CF1399 blastospore 38 (41/49, 15 sites, site 14 2/4) | Diploid | |
|  | CF1399 blastospore 39 (41/49, 15 sites, site 15 3/3) | Diploid | |
|  | CF1399 blastospore 40 (41/49, 15 sites, site 15 3/3) | Diploid | |
|  | CF1399 blastospore 41 (41/49, 15 sites, site 15 3/3) |  | |
| *pcl2*∆-2 | CF1535 blastospore 1 (22/40, 15 spots, spot2 4/5) | Diploid | |
|  | CF1535 blastospore 2 (22/40, 15 spots, spot2 4/5) |  | |
|  | CF1535 blastospore 3 (22/40, 15 spots, spot2 4/5) |  | |
|  | CF1535 blastospore 4 (22/40, 15 spots, spot2 4/5) | Diploid | |
|  | CF1535 blastospore 5 (22/40, 15 spots, spot3 1/3) | Diploid | |
|  | CF1535 blastospore 6 (22/40, 15 spots, spot5 1/3) | Diploid | |
|  | CF1535 blastospore 7 (22/40, 15 spots, spot6 2/3) | Diploid | |
|  | CF1535 blastospore 8 (22/40, 15 spots, spot6 2/3) | Diploid | |
|  | CF1535 blastospore 9 (22/40, 15 spots, spot7 4/4) | Diploid | |
|  | CF1535 blastospore 10 (22/40, 15 spots, spot7 4/4) |  | |
|  | CF1535 blastospore 11 (22/40, 15 spots, spot7 4/4) |  | |
|  | CF1535 blastospore 12 (22/40, 15 spots, spot7 4/4) | Diploid | |
|  | CF1535 blastospore 13 (22/40, 15 spots, spot9 1/3) | Diploid | |
|  | CF1535 blastospore 14 (22/40, 15 spots, spot11 3/3) | Diploid | |
|  | CF1535 blastospore 15 (22/40, 15 spots, spot11 3/3) |  | |
|  | CF1535 blastospore 16 (22/40, 15 spots, spot11 3/3) | Diploid | |
|  | CF1535 blastospore 17 (22/40, 15 spots, spot13 2/2) | Diploid | |
|  | CF1535 blastospore 18 (22/40, 15 spots, spot13 2/2) | Diploid | |
|  | CF1535 blastospore 19 (22/40, 15 spots, spot14 2/3) | Diploid | |
|  | CF1535 blastospore 20 (22/40, 15 spots, spot14 2/3) | Diploid | |
|  | CF1535 blastospore 21 (22/40, 15 spots, spot15 2/2) | Diploid | |
|  | CF1535 blastospore 22 (22/40, 15 spots, spot15 2/2) | Diploid | |
| *pcl6*∆-1 | CF1360 blastospore 1 (21/42, 10 sites, site 1 2/4) | Haploid/Diploid mix | |
|  | CF1360 blastospore 2 (21/42, 10 sites, site 1 2/4) | Haploid/Diploid mix | |
|  | CF1360 blastospore 3 (21/42, 10 sites, site 2 4/4) | Diploid | |
|  | CF1360 blastospore 4 (21/42, 10 sites, site 2 4/4) | Diploid | |
|  | CF1360 blastospore 5 (21/42, 10 sites, site 2 4/4) |  | |
|  | CF1360 blastospore 6 (21/42, 10 sites, site 2 4/4) |  | |
|  | CF1360 blastospore 7 (21/42, 10 sites, site 3 2/4) | Diploid | |
|  | CF1360 blastospore 8 (21/42, 10 sites, site 3 2/4) | Diploid | |
|  | CF1360 blastospore 9 (21/42, 10 sites, site 5 5/5) | Haploid/Diploid mix | |
|  | CF1360 blastospore 10 (21/42, 10 sites, site 5 5/5) | Haploid/Diploid mix | |
|  | CF1360 blastospore 11 (21/42, 10 sites, site 5 5/5) |  | |
|  | CF1360 blastospore 12 (21/42, 10 sites, site 5 5/5) |  | |
|  | CF1360 blastospore 13 (21/42, 10 sites, site 5 5/5) |  | |
|  | CF1360 blastospore 14 (21/42, 10 sites, site 7 5/5) | Haploid/Diploid mix | |
|  | CF1360 blastospore 15 (21/42, 10 sites, site 7 5/5) | Haploid/Diploid mix | |
|  | CF1360 blastospore 16 (21/42, 10 sites, site 7 5/5) |  | |
|  | CF1360 blastospore 17 (21/42, 10 sites, site 7 5/5) |  | |
|  | CF1360 blastospore 18 (21/42, 10 sites, site 7 5/5) |  | |
|  | CF1360 blastospore 19 (21/42, 10 sites, site 8 1/4) | Haploid/Diploid mix | |
|  | CF1360 blastospore 20 (21/42, 10 sites, site 10 2/3) | Diploid | |
|  | CF1360 blastospore 21 (21/42, 10 sites, site 10 2/3) | Diploid | |
| *pcl6*∆-2 | CF1361 blastospore 1 (28/42, 10 sites, site 1 2/5) | Haploid/Diploid mix | |
|  | CF1361 blastospore 2 (28/42, 10 sites, site 1 2/5) | Haploid/Diploid mix | |
|  | CF1361 blastospore 3 (28/42, 10 sites, site 3 2/4) | Haploid | |
|  | CF1361 blastospore 4 (28/42, 10 sites, site 3 2/4) | Haploid | |
|  | CF1361 blastospore 5 (28/42, 10 sites, site 4 5/5) | Haploid/Diploid mix | |
|  | CF1361 blastospore 6 (28/42, 10 sites, site 4 5/5) | Haploid/Diploid mix | |
|  | CF1361 blastospore 7 (28/42, 10 sites, site 4 5/5) |  | |
|  | CF1361 blastospore 8 (28/42, 10 sites, site 4 5/5) |  | |
|  | CF1361 blastospore 9 (28/42, 10 sites, site 4 5/5) |  | |
|  | CF1361 blastospore 10 (28/42, 10 sites, site 5 3/4) | Diploid | |
|  | CF1361 blastospore 11 (28/42, 10 sites, site 5 3/4) | Diploid | |
|  | CF1361 blastospore 12 (28/42, 10 sites, site 5 3/4) |  | |
|  | CF1361 blastospore 13 (28/42, 10 sites, site 6 4/4) | Haploid/Diploid mix | |
|  | CF1361 blastospore 14 (28/42, 10 sites, site 6 4/4) | Haploid/Diploid mix | |
|  | CF1361 blastospore 15 (28/42, 10 sites, site 6 4/4) |  | |
|  | CF1361 blastospore 16 (28/42, 10 sites, site 6 4/4) |  | |
|  | CF1361 blastospore 17 (28/42, 10 sites, site 7 4/4) | Haploid/Diploid mix | |
|  | CF1361 blastospore 18 (28/42, 10 sites, site 7 4/4) | Haploid/Diploid mix | |
|  | CF1361 blastospore 19 (28/42, 10 sites, site 7 4/4) |  | |
|  | CF1361 blastospore 20 (28/42, 10 sites, site 7 4/4) |  | |
|  | CF1361 blastospore 21 (28/42, 10 sites, site 8 1/7) | Diploid | |
|  | CF1361 blastospore 22 (28/42, 10 sites, site 9 4/5) | Haploid/Diploid mix | |
|  | CF1361 blastospore 23 (28/42, 10 sites, site 9 4/5) | Haploid/Diploid mix | |
|  | CF1361 blastospore 24 (28/42, 10 sites, site 9 4/5) |  | |
|  | CF1361 blastospore 25 (28/42, 10 sites, site 9 4/5) |  | |
|  | CF1361 blastospore 26 (28/42, 10 sites, site 10 3/3) | Diploid | |
|  | CF1361 blastospore 27 (28/42, 10 sites, site 10 3/3) | Diploid | |
|  | CF1361 blastospore 28 (28/42, 10 sites, site 10 3/3) |  | |
| *pcl9*∆-1 | CF1765 blastospore 1 (33/36, 12 spots, spot1 4/4) | Diploid | |
|  | CF1765 blastospore 2 (33/36, 12 spots, spot1 4/4) |  | |
|  | CF1765 blastospore 3 (33/36, 12 spots, spot1 4/4) |  | |
|  | CF1765 blastospore 4 (33/36, 12 spots, spot1 4/4) | Diploid | |
|  | CF1765 blastospore 5 (33/36, 12 spots, spot2 2/3) | Haploid | |
|  | CF1765 blastospore 6 (33/36, 12 spots, spot2 2/3) | Haploid | |
|  | CF1765 blastospore 7 (33/36, 12 spots, spot3 2/2) | Haploid | |
|  | CF1765 blastospore 8 (33/36, 12 spots, spot3 2/2) | Haploid | |
|  | CF1765 blastospore 9 (33/36, 12 spots, spot4 2/2) | Haploid | |
|  | CF1765 blastospore 10 (33/36, 12 spots, spot4 2/2) | Haploid | |
|  | CF1765 blastospore 11 (33/36, 12 spots, spot5 3/3) | Haploid | |
|  | CF1765 blastospore 12 (33/36, 12 spots, spot5 3/3) |  | |
|  | CF1765 blastospore 13 (33/36, 12 spots, spot5 3/3) | Haploid | |
|  | CF1765 blastospore 14 (33/36, 12 spots, spot6 3/3) | Haploid | |
|  | CF1765 blastospore 15 (33/36, 12 spots, spot6 3/3) |  | |
|  | CF1765 blastospore 16 (33/36, 12 spots, spot6 3/3) | Haploid | |
|  | CF1765 blastospore 17 (33/36, 12 spots, spot7 2/3) | Haploid | |
|  | CF1765 blastospore 18 (33/36, 12 spots, spot7 2/3) | Haploid | |
|  | CF1765 blastospore 19 (33/36, 12 spots, spot8 4/4) | Haploid | |
|  | CF1765 blastospore 20 (33/36, 12 spots, spot8 4/4) |  | |
|  | CF1765 blastospore 21 (33/36, 12 spots, spot8 4/4) |  | |
|  | CF1765 blastospore 22 (33/36, 12 spots, spot8 4/4) | Haploid | |
|  | CF1765 blastospore 23 (33/36, 12 spots, spot9 1/2) | Haploid | |
|  | CF1765 blastospore 24 (33/36, 12 spots, spot10 4/4) | Diploid | |
|  | CF1765 blastospore 25 (33/36, 12 spots, spot10 4/4) |  | |
|  | CF1765 blastospore 26 (33/36, 12 spots, spot10 4/4) |  | |
|  | CF1765 blastospore 27 (33/36, 12 spots, spot10 4/4) | Diploid | |
|  | CF1765 blastospore 28 (33/36, 12 spots, spot 11 3/3) | Haploid | |
|  | CF1765 blastospore 29 (33/36, 12 spots, spot 11 3/3) |  | |
|  | CF1765 blastospore 30 (33/36, 12 spots, spot 11 3/3) | Haploid | |
|  | CF1765 blastospore 31 (33/36, 12 spots, spot 12 3/3) | Haploid | |
|  | CF1765 blastospore 32 (33/36, 12 spots, spot 12 3/3) |  | |
|  | CF1765 blastospore 33 (33/36, 12 spots, spot 12 3/3) | Haploid | |
| *pcl9*∆-2 | CF1768 blastospore 1 (30/31, 12 spots, spot1 2/2) | Haploid | |
|  | CF1768 blastospore 2 (30/31, 12 spots, spot1 2/2) | Haploid | |
|  | CF1768 blastospore 3 (30/31, 12 spots, spot2 2/2) | Haploid | |
|  | CF1768 blastospore 4 (30/31, 12 spots, spot2 2/2) | Haploid | |
|  | CF1768 blastospore 5 (30/31, 12 spots, spot3 3/3) | Haploid | |
|  | CF1768 blastospore 6 (30/31, 12 spots, spot3 3/3) |  | |
|  | CF1768 blastospore 7 (30/31, 12 spots, spot3 3/3) | Haploid | |
|  | CF1768 blastospore 8 (30/31, 12 spots, spot4 3/3) | Diploid | |
|  | CF1768 blastospore 9 (30/31, 12 spots, spot4 3/3) |  | |
|  | CF1768 blastospore 10 (30/31, 12 spots, spot4 3/3) | Diploid | |
|  | CF1768 blastospore 11 (30/31, 12 spots, spot5 2/3) | Haploid | |
|  | CF1768 blastospore 12 (30/31, 12 spots, spot5 2/3) | Haploid | |
|  | CF1768 blastospore 13 (30/31, 12 spots, spot6 3/3) | Haploid | |
|  | CF1768 blastospore 14 (30/31, 12 spots, spot6 3/3) |  | |
|  | CF1768 blastospore 15 (30/31, 12 spots, spot6 3/3) | Haploid | |
|  | CF1768 blastospore 16 (30/31, 12 spots, spot7 2/2) | Diploid | |
|  | CF1768 blastospore 17 (30/31, 12 spots, spot7 2/2) | Diploid | |
|  | CF1768 blastospore 18 (30/31, 12 spots, spot8 3/3) | Haploid | |
|  | CF1768 blastospore 19 (30/31, 12 spots, spot8 3/3) |  | |
|  | CF1768 blastospore 20 (30/31, 12 spots, spot8 3/3) | Haploid | |
|  | CF1768 blastospore 21 (30/31, 12 spots, spot9 3/3) | Diploid | |
|  | CF1768 blastospore 22 (30/31, 12 spots, spot9 3/3) |  | |
|  | CF1768 blastospore 23 (30/31, 12 spots, spot9 3/3) | Diploid | |
|  | CF1768 blastospore 24 (30/31, 12 spots, spot10 3/3) | Haploid | |
|  | CF1768 blastospore 25 (30/31, 12 spots, spot10 3/3) |  | |
|  | CF1768 blastospore 26 (30/31, 12 spots, spot10 3/3) | Haploid | |
|  | CF1768 blastospore 27 (30/31, 12 spots, spot11 2/2) | Haploid | |
|  | CF1768 blastospore 28 (30/31, 12 spots, spot11 2/2) | Haploid | |
|  | CF1768 blastospore 29 (30/31, 12 spots, spot12 2/2) | Haploid | |
|  | CF1768 blastospore 30 (30/31, 12 spots, spot12 2/2) | Haploid | |
| *cks1*∆-1 | CF1367 blastospore 1 (21/40, 10 sites, site 3 3/4) | Haploid | |
|  | CF1367 blastospore 2 (21/40, 10 sites, site 3 3/4) | Haploid | |
|  | CF1367 blastospore 3 (21/40, 10 sites, site 3 3/4) |  | |
|  | CF1367 blastospore 4 (21/40, 10 sites, site 5 1/4) | Haploid | |
|  | CF1367 blastospore 5 (21/40, 10 sites, site 6 4/4) | Haploid | |
|  | CF1367 blastospore 6 (21/40, 10 sites, site 6 4/4) | Haploid | |
|  | CF1367 blastospore 7 (21/40, 10 sites, site 6 4/4) |  | |
|  | CF1367 blastospore 8 (21/40, 10 sites, site 6 4/4) |  | |
|  | CF1367 blastospore 9 (21/40, 10 sites, site 7 3/3) | Haploid | |
|  | CF1367 blastospore 10 (21/40, 10 sites, site 7 3/3) | Haploid | |
|  | CF1367 blastospore 11 (21/40, 10 sites, site 7 3/3) |  | |
|  | CF1367 blastospore 12 (21/40, 10 sites, site 8 4/4) | Haploid | |
|  | CF1367 blastospore 13 (21/40, 10 sites, site 8 4/4) | Haploid | |
|  | CF1367 blastospore 14 (21/40, 10 sites, site 8 4/4) |  | |
|  | CF1367 blastospore 15 (21/40, 10 sites, site 8 4/4) |  | |
|  | CF1367 blastospore 16 (21/40, 10 sites, site 9 2/4) | Haploid | |
|  | CF1367 blastospore 17 (21/40, 10 sites, site 9 2/4) | Haploid | |
|  | CF1367 blastospore 18 (21/40, 10 sites, site 10 4/5) | Haploid | |
|  | CF1367 blastospore 19 (21/40, 10 sites, site 10 4/5) | Haploid | |
|  | CF1367 blastospore 20 (21/40, 10 sites, site 10 4/5) |  | |
|  | CF1367 blastospore 21 (21/40, 10 sites, site 10 4/5) |  | |
| *cks1*∆-2 | CF1414 blastospore 1 (21/39, 11 sites, site1 5/5) | Haploid | |
|  | CF1414 blastospore 2 (21/39, 11 sites, site1 5/5) | Haploid | |
|  | CF1414 blastospore 3 (21/39, 11 sites, site1 5/5) |  | |
|  | CF1414 blastospore 4 (21/39, 11 sites, site1 5/5) |  | |
|  | CF1414 blastospore 5 (21/39, 11 sites, site1 5/5) |  | |
|  | CF1414 blastospore 6 (21/39, 11 sites, site2 3/4) | Haploid | |
|  | CF1414 blastospore 7 (21/39, 11 sites, site2 3/4) | Haploid | |
|  | CF1414 blastospore 8 (21/39, 11 sites, site2 3/4) |  | |
|  | CF1414 blastospore 9 (21/39, 11 sites, site3 3/4) | Haploid | |
|  | CF1414 blastospore 10 (21/39, 11 sites, site3 3/4) | Haploid | |
|  | CF1414 blastospore 11 (21/39, 11 sites, site3 3/4) |  | |
|  | CF1414 blastospore 12 (21/39, 11 sites, site6 1/3) | Haploid | |
|  | CF1414 blastospore 13 (21/39, 11 sites, site7 1/2) | Haploid | |
|  | CF1414 blastospore 14 (21/39, 11 sites, site8 3/4) | Haploid | |
|  | CF1414 blastospore 15 (21/39, 11 sites, site8 3/4) | Haploid | |
|  | CF1414 blastospore 16 (21/39, 11 sites, site8 3/4) |  | |
|  | CF1414 blastospore 17 (21/39, 11 sites, site9 2/3) | Haploid | |
|  | CF1414 blastospore 18 (21/39, 11 sites, site9 2/3) | Haploid | |
|  | CF1414 blastospore 19 (21/39, 11 sites, site10 2/4) | Haploid | |
|  | CF1414 blastospore 20 (21/39, 11 sites, site10 2/4) | Haploid | |
|  | CF1414 blastospore 21 (21/39, 11 sites, site11 1/2) | Haploid | |
| *cks2*∆-1 | CF1379 blastospore 1 (44/57, 10 sites, site 1 7/8) | Diploid | |
|  | CF1379 blastospore 2 (44/57, 10 sites, site 1 7/8) | Diploid | |
|  | CF1379 blastospore 3 (44/57, 10 sites, site 1 7/8) |  | |
|  | CF1379 blastospore 4 (44/57, 10 sites, site 1 7/8) |  | |
|  | CF1379 blastospore 5 (44/57, 10 sites, site 1 7/8) |  | |
|  | CF1379 blastospore 6 (44/57, 10 sites, site 1 7/8) |  | |
|  | CF1379 blastospore 7 (44/57, 10 sites, site 1 7/8) |  | |
|  | CF1379 blastospore 8 (44/57, 10 sites, site 2 2/6) | Diploid | |
|  | CF1379 blastospore 9 (44/57, 10 sites, site 2 2/6) | Diploid | |
|  | CF1379 blastospore 10 (44/57, 10 sites, site 4 3/5) | Diploid | |
|  | CF1379 blastospore 11 (44/57, 10 sites, site 4 3/5) | Diploid | |
|  | CF1379 blastospore 12 (44/57, 10 sites, site 4 3/5) |  | |
|  | CF1379 blastospore 13 (44/57, 10 sites, site 5 7/7) | Haploid | |
|  | CF1379 blastospore 14 (44/57, 10 sites, site 5 7/7) | Haploid | |
|  | CF1379 blastospore 15 (44/57, 10 sites, site 5 7/7) |  | |
|  | CF1379 blastospore 16 (44/57, 10 sites, site 5 7/7) |  | |
|  | CF1379 blastospore 17 (44/57, 10 sites, site 5 7/7) |  | |
|  | CF1379 blastospore 18 (44/57, 10 sites, site 5 7/7) |  | |
|  | CF1379 blastospore 19 (44/57, 10 sites, site 5 7/7) |  | |
|  | CF1379 blastospore 20 (44/57, 10 sites, site 6 4/5) | Haploid | |
|  | CF1379 blastospore 21 (44/57, 10 sites, site 6 4/5) | Haploid | |
|  | CF1379 blastospore 22 (44/57, 10 sites, site 6 4/5) |  | |
|  | CF1379 blastospore 23 (44/57, 10 sites, site 6 4/5) |  | |
|  | CF1379 blastospore 24 (44/57, 10 sites, site 7 4/5) | Haploid | |
|  | CF1379 blastospore 25 (44/57, 10 sites, site 7 4/5) | Haploid | |
|  | CF1379 blastospore 26 (44/57, 10 sites, site 7 4/5) |  | |
|  | CF1379 blastospore 27 (44/57, 10 sites, site 7 4/5) |  | |
|  | CF1379 blastospore 28 (44/57, 10 sites, site 8 5/5) | Haploid | |
|  | CF1379 blastospore 29 (44/57, 10 sites, site 8 5/5) | Haploid | |
|  | CF1379 blastospore 30 (44/57, 10 sites, site 8 5/5) |  | |
|  | CF1379 blastospore 31 (44/57, 10 sites, site 8 5/5) |  | |
|  | CF1379 blastospore 32 (44/57, 10 sites, site 8 5/5) |  | |
|  | CF1379 blastospore 33 (44/57, 10 sites, site 9 6/6) | Haploid | |
|  | CF1379 blastospore 34 (44/57, 10 sites, site 9 6/6) | Haploid | |
|  | CF1379 blastospore 35 (44/57, 10 sites, site 9 6/6) |  | |
|  | CF1379 blastospore 36 (44/57, 10 sites, site 9 6/6) |  | |
|  | CF1379 blastospore 37 (44/57, 10 sites, site 9 6/6) |  | |
|  | CF1379 blastospore 38 (44/57, 10 sites, site 9 6/6) |  | |
|  | CF1379 blastospore 39 (44/57, 10 sites, site 10 6/6) | Diploid | |
|  | CF1379 blastospore 40 (44/57, 10 sites, site 10 6/6) | Diploid | |
|  | CF1379 blastospore 41 (44/57, 10 sites, site 10 6/6) |  | |
|  | CF1379 blastospore 42 (44/57, 10 sites, site 10 6/6) |  | |
|  | CF1379 blastospore 43 (44/57, 10 sites, site 10 6/6) |  | |
|  | CF1379 blastospore 44 (44/57, 10 sites, site 10 6/6) |  | |
| *cks2*∆-2 | CF1387 blastospore 1 (40/53, 10 sites, site 1 4/4) | Haploid | |
|  | CF1387 blastospore 2 (40/53, 10 sites, site 1 4/4) | Haploid | |
|  | CF1387 blastospore 3 (40/53, 10 sites, site 1 4/4) |  | |
|  | CF1387 blastospore 4 (40/53, 10 sites, site 1 4/4) |  | |
|  | CF1387 blastospore 5 (40/53, 10 sites, site 3 3/4) | Haploid | |
|  | CF1387 blastospore 6 (40/53, 10 sites, site 3 3/4) | Haploid | |
|  | CF1387 blastospore 7 (40/53, 10 sites, site 3 3/4) |  | |
|  | CF1387 blastospore 8 (40/53, 10 sites, site 4 5/5) | Diploid | |
|  | CF1387 blastospore 9 (40/53, 10 sites, site 4 5/5) | Diploid | |
|  | CF1387 blastospore 10 (40/53, 10 sites, site 4 5/5) |  | |
|  | CF1387 blastospore 11 (40/53, 10 sites, site 4 5/5) |  | |
|  | CF1387 blastospore 12 (40/53, 10 sites, site 4 5/5) |  | |
|  | CF1387 blastospore 13 (40/53, 10 sites, site 5 5/6) | Haploid | |
|  | CF1387 blastospore 14 (40/53, 10 sites, site 5 5/6) | Haploid | |
|  | CF1387 blastospore 15 (40/53, 10 sites, site 5 5/6) |  | |
|  | CF1387 blastospore 16 (40/53, 10 sites, site 5 5/6) |  | |
|  | CF1387 blastospore 17 (40/53, 10 sites, site 5 5/6) |  | |
|  | CF1387 blastospore 18 (40/53, 10 sites, site 6 5/5) | Haploid | |
|  | CF1387 blastospore 19 (40/53, 10 sites, site 6 5/5) | Haploid | |
|  | CF1387 blastospore 20 (40/53, 10 sites, site 6 5/5) |  | |
|  | CF1387 blastospore 21 (40/53, 10 sites, site 6 5/5) |  | |
|  | CF1387 blastospore 22 (40/53, 10 sites, site 6 5/5) |  | |
|  | CF1387 blastospore 23 (40/53, 10 sites, site 7 5/5) | Diploid | |
|  | CF1387 blastospore 24 (40/53, 10 sites, site 7 5/5) | Diploid | |
|  | CF1387 blastospore 25 (40/53, 10 sites, site 7 5/5) |  | |
|  | CF1387 blastospore 26 (40/53, 10 sites, site 7 5/5) |  | |
|  | CF1387 blastospore 27 (40/53, 10 sites, site 7 5/5) |  | |
|  | CF1387 blastospore 28 (40/53, 10 sites, site 8 2/4) | Diploid | |
|  | CF1387 blastospore 29 (40/53, 10 sites, site 8 2/4) | Diploid | |
|  | CF1387 blastospore 30 (40/53, 10 sites, site 9 5/5) | Diploid | |
|  | CF1387 blastospore 31 (40/53, 10 sites, site 9 5/5) | Diploid | |
|  | CF1387 blastospore 32 (40/53, 10 sites, site 9 5/5) |  | |
|  | CF1387 blastospore 33 (40/53, 10 sites, site 9 5/5) |  | |
|  | CF1387 blastospore 34 (40/53, 10 sites, site 9 5/5) |  | |
|  | CF1387 blastospore 35 (40/53, 10 sites, site 10 6/8) | Diploid | |
|  | CF1387 blastospore 36 (40/53, 10 sites, site 10 6/8) | Diploid | |
|  | CF1387 blastospore 37 (40/53, 10 sites, site 10 6/8) |  | |
|  | CF1387 blastospore 38 (40/53, 10 sites, site 10 6/8) |  | |
|  | CF1387 blastospore 39 (40/53, 10 sites, site 10 6/8) |  | |
|  | CF1387 blastospore 40 (40/53, 10 sites, site 10 6/8) |  | |
| *P_GAL7_-CLB3* | CF1715 blastospore 1 (3/40, 15 sites, site1 2/3) germinated on YPG | Haploid | |
|  | CF1715 blastospore 2 (3/40, 15 sites, site1 2/3) germinated on YPG | Haploid | |
|  | CF1715 blastospore 3 (3/40, 15 sites, site10 1/4) germinated on YPG | Haploid | |
| **Plasmid** | **Genotype** | **Sources** | |
| pAG32 | *HYG AMP* | [4] | |
| pAI3 | *NAT AMP* | [5] | |
| pJAF1 | *NEO AMP* | [6] | |
| pJAF15 | *HYG AMP* | [6] | |
| pXL1 | *P_GPD1_* *NEO* *KAN AMP* | [7] | |
| pXL1-Cas9-HygB | *CAS9-HYGB* | [8] | |
| pYF515 | *sgRNA* scaffold | [9] | |
| pSDMA25 | *SH-NAT AMP* (*C. neoformans*) | [10] | |
| pCF3 | *SH-NEO AMP* (*C. deneoforman*) | This study | |
| pNURAT | *NURAT* in pAI3 | This study | |
| pCF7 | pNURAT in pCF3 | This study | |
| pSH5 | *P_GAL7_-CLB3-NEO KAN AMP* | This study | |

* Blastospore information (survival of all blastospores dissected for the given strain, total number of sites, the site that the blastospore is derived from, survival of blastospores dissected from this site).

** For each budding site, no more than two blastospores were chosen for FACS determination of ploidy.

**Reference**

1. Lin, X., Hull, C.M., and Heitman, J. (2005). Sexual reproduction between partners of the same mating type in *Cryptococcus neoformans*. Nature *434*, 1017-1021.

2. Zhai, B., Zhu, P., Foyle, D., Upadhyay, S., Idnurm, A., and Lin, X. (2013). Congenic strains of the filamentous form of *Cryptococcus neoformans* for studies of fungal morphogenesis and virulence. Infect. Immun. *81*, 2626-2637.

3. Ni, M., Feretzaki, M., Li, W., Floyd-Averette, A., Mieczkowski, P., Dietrich, F.S., et al. (2013). Unisexual and heterosexual meiotic reproduction generate aneuploidy and phenotypic diversity *de novo* in the yeast *Cryptococcus neoformans*. PLoS Biol. *11*, e1001653.

4. Goldstein, A.L., and McCusker, J.H. (1999). Three new dominant drug resistance cassettes for gene disruption in *Saccharomyces cerevisiae*. Yeast *15*, 1541-1553.

5. Idnurm, A., Reedy, J.L., Nussbaum, J.C., and Heitman, J. (2004). *Cryptococcus neoformans* virulence gene discovery through insertional mutagenesis. Eukaryot. Cell *3*, 420-429.

6. Fraser, J.A., Subaran, R.L., Nichols, C.B., and Heitman, J. (2003). Recapitulation of the sexual cycle of the primary fungal pathogen *Cryptococcus neoformans* var. *gattii*: implications for an outbreak on Vancouver Island, Canada. Eukaryot. Cell *2*, 1036-1045.

7. Hsueh, Y.P., Xue, C., and Heitman, J. (2009). A constitutively active GPCR governs morphogenic transitions in *Cryptococcus neoformans*. EMBO J. *28*, 1220-1233.

8. Fan, Y., and Lin, X. (2018). Multiple applications of a transient CRISPR-Cas9 coupled with electroporation (TRACE) Ssystem in the *Cryptococcus neoformans* species complex. Genetics *208*, 1357-1372.

9. Fang, Y., Cui, L., Gu, B., Arredondo, F., and Tyler, B.M. (2017). Efficient genome editing in the oomycete *Phytophthora sojae* using CRISPR/Cas9. Curr. Protoc. Microbiol. *44*, 21A 21 21-21A 21 26.

10. Arras, S.D., Chitty, J.L., Blake, K.L., Schulz, B.L., and Fraser, J.A. (2015). A genomic safe haven for mutant complementation in *Cryptococcus neoformans*. PLoS ONE *10*, e0122916.
