# Supplementary material for: Dynamic genome plasticity during unisexual reproduction in the human fungal pathogen Cryptococcus deneoformans
Source: PLoS Genet. 2021 Nov 29;17(11):e1009935. doi: 10.1371/journal.pgen.1009935 (PMC8670703; doi:10.1371/journal.pgen.1009935)
Supplement: Attachment — Submitted filename: Response to reviewers.docx [file pgen.1009935.s019.docx]

Reviewers’ comments are shown in blue, and changes made in response to each of the comments are shown in black immediately after the reviewer’s comment.

**Reviewer #1:** To investigate the functions underlying ploidy change during unisexual reproduction in C. deneoformans, Fu et al. first identified cell cycle-regulating genes encoding cyclins, cyclin-dependent kinases (CDK), and CDK regulators. They subsequently identified four cyclin genes and two CDK regulator genes for focused analysis based differential expression during unisexual reproduction. In general, they identified genes that contributed to diploidization. They also developed and employed a NAT-URA construct to monitor ploidy changes during unisexual reproduction and to detect segmental aneuploidy. Overall, the results in the manuscript provide an interesting illustration of aspects of genome plasticity (including segmental aneuploidy) for an important fungal pathogen in the context of cell cycle regulating functions and unisexual reproduction. For the most part, the data are clearly presented and support the conclusions. I only have a few minor issues to point out.

Thank you for this kind and thoughtful critique!

Lines 193-198. I found this description of the expression analysis to be somewhat confusing with regard to the description of the different behavior of PCL2. It is unclear from the description why this gene was different. Can a bit more detail be provided to better explain the difference?

We agree that the data presentation may be confusing for *PCL2*. To clarify this issue, we replotted the expression levels to show the expression dynamics on V8 medium and removed all background black lines to simplify the data presentation. A dashed black line was drawn to indicate the time point assayed in this study. *PCL2* is regulated differently compared to *CLB3* and *CKS2* as the gene was first upregulated at the 12-hour time point and then down regulated at later time points. We revised the text from line 198 to line 204 to clarify this observation “In agreement with our findings, all six genes were previously shown to be differentially expressed after growth on V8 medium for 12, 24, and 48 hours compared to growth on YPD for 12 hours (Figure S1B). *PCL6*, *PCL9*, and *CKS1* expression levels peaked at either the 24- or the 48-hour time point on V8 medium (Figure S1B). *CLB3* and *CKS2* were down-regulated on V8 medium, while *PCL2* had an initial upregulation on V8 at the 12-hour time point and then was down-regulated at later time points (Figure S1B) [39].”

Line 275-277. Something is wrong with construction of this sentence.

We revised the sentence to “More than 50% of *pcl6*∆ blastospores produced mixed populations of haploid and diploid cells, while about 80% of the *pcl9*∆ and 50% of the *cks*2∆ blastospores were haploid (Figure 2 and Table S2). Deletion of *CKS1* and suppressed expression of *CLB3* led to the production of only haploid blastospores (Figure 2 and Table S2).”

Line 283. Unclear phrase “more than half of the pcl6∆ blastospores”

We revised the sentence to “To further decipher the mixed haploid and diploid cell populations observed among the pcl6∆ blastospores and determine if *PCL6* is required for diploid maintenance, we streaked cells derived from diploid wild-type, diploid pcl6∆, and mixed haploid and diploid *pcl6*∆ blastospores for single colonies and subsequently determined their ploidy (Table S3)”

Line 388. …tested were found to be resistant

We corrected the text as recommended.

**Reviewer #2:** Fu et al., observe the impact of cyclin and cyclin dependent kinases on ploidy during growth in rich media and mating media (V8) over time, leveraging the ability of C. deneoformans to undergo unisexual reproduction to study genetic roles during yeast-phase growth and blastospore production. The authors report increased expression of PCL9, PCL6, and CKS1 and decreased expression of PCL2 and CLB3 under mating conditions. Using single mutants, they identify CKS1 and CLB3 as having an impact on spore production and also observe that on V8, mutants were defective for blastospores, with low germination and different ploidy (haploid or diploid) compared to the parental control (diploid, rare aneuploid). Using flow cytometry and a Nat/Ura (NURAT) reporter sensitive to homologous recombination and segmental duplication/diploidy, they find that growth on V8 alters cell ploidy and recombination rates. Interestingly, they also observe high levels of aneuploidy/segmental duplication in areas of the genome relevant for drug resistance (Ch1) during yeast-phase growth, and highlight a role for their genes of interest in regulating this process.

Overall, the authors have raised the possibility that there is a mating-specific mechanism for ploidy increase that is regulated by a mating-specific suite of cyclin and CDKs in C. deneoformans. Despite these interesting findings, it remains unclear what, if any, direct role these genes are playing in regulating ploidy, aneuploidy, or homologous recombination, how these genes interact with each other, why they are differentially regulated, or their shared or divergent targets. It is already well established that cyclins impact ploidy in a variety of systems, so it’s not surprising that the cyclins in C. deneoformans will do the same.
Some further insight into the specific role of cyclins in this process would be of general interest. For example, in the discussion, the authors raise a number of hypotheses about regulation of Pho85, but do not test a pho85 mutant for impacts on genome stability or investigate its potential as a target for their cyclins of interest. Alternately, why would there be a higher requirement for Glc7-regulation by Pcl6 during V8 growth than during rich-media growth? Can the authors identify a specific Cks1 target to support their hypothesis that Cks1 and Cks2 are functionally divergent rather than just differentially expressed?

We appreciate the reviewer raising these insightful questions. We are very interested in further exploring the interactomes for these six cell-cycle-regulating genes and studying mechanisms as to how these genes may regulate homologous recombination, cell cycle, and ploidy transition during unisexual reproduction. However, we respectfully suggest that such studies are worthy of future concerted effort and are beyond the scope of the current study. To hypothesize how these cell cycle regulators could contribute to ploidy transition, we added two examples illustrating how cyclins contribute to polyploid cell formation in *S. cerevisiae* and *C. neoformans* from line 149 to line 151 in introduction and from line 584 to line 593 in discussion, and discussed the roles of the cell cycle regulating genes in cell cycle progression referencing Kelliher and colleagues’ study on the cell cycle in *C. neoformans* from line 567 to line 583.

Overall, the manuscript is well written, and figures are clear and concise. There are some small points where additional detail is needed for methodology (see minor comments below).

Line 258: for CLB3, it would be better to see the S5B data brought into the main text.

We moved Figure S5B to Figure 1C.

This finding of V8 altering expression of cell cycle genes should be placed in the context of cell cycle progression. The authors should consider citing Kelliher et al as a key paper comparing S. cerevisae cell cycle and C. neoformans under rich media conditions. However, altered expression of these genes on different media is consistent with previous findings (which the authors point out and cite).

We apologize for overlooking this important work in our original manuscript and thank the reviewer for bringing it to our attention. We have now revised the manuscript to include discussion of this work in relation to the cell cycle-regulating genes studied in our paper from line 567 to line 583.

For statistical analysis, in some instances it appears the authors are reporting p-values using Student’s t-test without correcting for repeated testing (e.g., Figure 5A, mutants vs WT). If true, this is not appropriate. The authors must correct for multiple comparisons.

Thank you for bringing our attention to this important statistical issue. We have corrected the student’s t-test with Bonferroni correction for data shown in Figure 5 and Figure S11.

Flow cytometry gating strategy to exclude doublets must be described and a representative scatterplot included in the supplemental material.

We included representative images showing the gating strategy to exclude doublets in revised Figure 2.

Figure S3 DAPI staining hasn’t been particularly effective. It would be better to use a nuclear dye that fluoresces in a different wavelength from CFW so the reader can differentiate CFW staining of septa, bud scars, and inappropriate chitin deposition from nuclear material.

We agree the DAPI staining is less effective compared to propidium iodide staining, especially when staining together with CFW. However, our main objective in Figure S3 is to show elongated cellular morphology. To highlight this, we added dashed white lines to outline representative elongated cells in *cks1*∆ mutant and *P_GAL7_-CLB3* YPD panels.

Line 135: these references aren’t strictly focused on C. deneoformans ploidy stimuli, but instead show the impact of specific stimuli (density, QSP, etc.) on C. neoformans. There are others that would be a better fit, including work by Zhao et al., 2020 in Current Biology.

We thank the reviewer for this excellent suggestion. We have added reference of the Zhao et al paper as recommended. We also agree that the cited references have a broader focus than just ploidy transition, but we do think they provide evidence that cell density and quorum sensing molecules impact ploidy transition during unisexual reproduction, thus we have opted to retain these references.

Line 287: this is just a bit confusing. “all single colonies derived from diploid blastospores were diploid, and only one single colony derived from mixed haploid-diploid blastospores remained a mixed-ploidy population. All other single colonies from the mixed 1N/2N blastospores were either haploid or diploid”
Does this mean “all single colonies derived from diploid pcl6 blastospores were diploid, and only one single colony derived from mixed pcl6 haploid-diploid blastospores remained a mixed-ploidy population. All other single colonies from the mixed pcl6 1N/2N blastospores were either haploid or diploid”
or
“all single colonies derived from both WT and pcl6 diploid blastospores were diploid, and only one single colony derived from mixed plc6 haploid-diploid blastospores remained a mixed-ploidy population. All other single colonies from the mixed pcl6 1N/2N blastospores were either haploid or diploid”

I know it’s functionally the same thing in terms of data interpretation, but it’s just a bit of a slog for the reader and could be clarified.

We appreciate the reviewer’s suggestion and have revised the text from line 292 to line 301 to make it clearer.

Line 480: what is meant by “sufficient”? Is this supported by a power calculation, or just a matter of convenience?

We meant to compare mutants that yielded comparable number of NAT^R^, Ura^+^ colonies to wild type. To avoid confusion, we changed “sufficient” to “comparable”.

The references for use of HU and nocodozole for arrest at G1/S and G2/M phase don’t actually show that these drugs promote cell cycle arrest per se. This is a bit misleading. References to these techniques in other yeast, where they are well established, would be more appropriate. To their credit, the data the authors present are the best proof I’ve seen that these tools are of use in C. deneoformans.

Thank you for the kind acknowledgement and recommendation on references. We included two references utilizing HU (PMID: 4120066) and nocodazole (PMID: 3049620) for G1/S and G2/M arrests in *Saccharomyces cerevisiae*.

**Reviewer #3:** The manuscript by Fu and colleagues explores the 6 cyclins, cyclin-dependent kinases, and other CDK regulatory genes with differential expression during unisexual reproduction in the Cryptococcus deneoformans strain XL280 using a mutagenesis approach. The authors also develop a fate mapping system for C. deneoformans to detect homologous recombination and the presence of locus duplications in the genome associated with unisexual reproduction associated, which the authors refer to as NURAT. While the manuscript presents interesting data, and is well-written, there are a number of concerns related to data presentation and analysis, whether the NURAT system can be used to for the purposes the authors propose, and whether the conclusions drawn are appropriate given the limited experimental data presented. Detailed concerns are as follows:

Major Concerns:

1. Given that the authors analyzed the phenotypes of the mutants in unisexual mating in Figure 1, and then in the yeast form in Figures S3-S5, the authors also need to analyze the mutants for their phenotype in bisexual mating. This is particularly pertinent given that many of the mutants show defects in unisexual mating associated with hyphal growth and sporulation – both of which are also critical in bisexual mating and thus it is important to know if these cyclins are utilized in both of the mating systems or are only used during unisexual mating. This point is critical for the overall conclusions of the manuscript as the authors often make statements referring only to unisexual mating, while at other time they make broad generalizations and the reasoning for this is often not transparent.

Thank you for pointing out this important issue and we agree that bisexual mating phenotypes for these mutants are of interest. We performed bisexual mating assays for the deletion mutants and confirmed that deletion of these cell cycle regulating genes had a smaller impact on bisexual reproduction, further confirming our hypothesis that expression of these genes was coordinated during unisexual reproduction. The images for bisexual mating are provided in Figure S2B and we described these results from line 225 to line 229. Different from *cks1*∆ mutants, *clb3*∆ mutants displayed a defect in filamentation and sporulation phenotypes during bisexual reproduction similar to unisexual reproduction as previously reported by Pengjie Hu and colleagues in (PMID: 33165808), suggesting that Clb3 plays a pivotal role during sporogenesis in both unisexual and bisexual reproduction. We discussed this finding in discussion from line 545 to line 549.

2. The NURAT system is not well described. For an individual unfamiliar with the system, and particularly its interaction with homologous recombination, the description is very difficult to comprehend. Questions that arise are: When is homologous recombination occurring? During integration of the construct into the genome? During mitosis? During meiosis? How can you differentiate between homologous recombination occurring during mitosis and during meiosis in this system? Why wouldn’t two short versions of the NURAT construct be produced in diploids or polyploids if they undergo multiple rounds of endoreplication? Why didn’t you detect any of these?

Thank you for pointing out this issue. We reorganized Figure 3A to show a better schematic diagram for the *NURAT* system. Detection of copy number increase utilizing *NURAT* cassette requires normal homologous recombination function during either mitosis or meiosis. In order to select colonies with increased copy number, we screened colonies that were both NAT resistant and uracil prototrophic, thus we were not able to detect diploid colonies with two short versions of the *NURAT* cassette. We revised the text from line 322 to line 334 to better describe the *NURAT* system.

3. While I believe I understand why the diploid NURAT strains were originally developed as a mechanism to verify the NURAT construct was working as expected, I do not understand why the authors then used these strains in Figure 3B when they were testing their experimental hypothesis. It is not apparent to me what relevance performing these studies in a diploid has to the overall focus of the studies or their conclusions. Rather, the utilization of the diploid strain may inadvertently lead the authors to draw inappropriate conclusions about ploidy using these artificial diploids. In other fungal species such as S. cerevisiae strains with artificially increased ploidy have previously been shown to have increased rates of genome modifications similar to those the authors observed.

We apologize for this confusion. Given this is the first report of utilizing the *NURAT* reporter in detecting ploidy increase, we are interested in the robustness of *NURAT* reporter in differentiating cells with different ploidy levels. To reflect this point, we revised the sentence from line 335 to line 338: “To test the robustness of the *NURAT* reporter in detecting diploid cells versus haploid cells, we generated two diploid *NURAT*/*NURAT* strains through blastospore dissection of the haploid *NURAT* strain (Figure S6) and performed fluctuation assays (Figure S7)”.

4. Based on the red/blue designations in the legend of Figure 4 it is unclear whether the authors have sufficient read depth to determine how many copies of each of these regions are present. 2 copies? 4 copies? 10 copies?

Based on the FACS determination of colonies’ ploidy (Figure 4A) and read depth across the whole segmentally duplicated regions (Figure 4A and 4B) and the reads aligned to the boundary of these regions (Figure S9), these regions are present with one extra copy compared to the rest of the genome. We clarified this issue on Line 388.

5. Genomic analysis of segmental aneuploidy sequencing was only performed on multiple blastospore derived segmental aneuploidies, and not on any of the diploid isolates identified in the yeast cultures. Yet the authors conclude that their results are both specific to unisexual mating AND will be a generalizable trait in Cryptococcus. The authors need to sequence multiple diploid isolates from the yeast cultures (not just the aneuploid blastospores) to determine if segmental aneuploidies are also present in these strains, and if so, whether they show similar characteristics.

The reviewer’s point is well taken, and we acknowledge that the evidence provided in the current study is not sufficient to support the generalization of segmental aneuploidy in Cryptococcus, which is not the main objective of this study. To avoid overstatement, we concluded the discussion to indicate that future examination of the prevalence of segmental aneuploidy in different isolates and strains with different ploidy status is required to understand its biological significance in *Cryptococcus*.

6. While the authors show an association of the transposons with the observed segmental aneuploidies, and therefore postulate the transposons contribute to aneuploidy formation in this region, the authors do ot provide any experimental proof to support this claim. For example, if the transposon is important for formation of the aneuploidy in this region, then deletion of the transposon should result in a reduction in aneuploidy formation at this site and/or extension of the aneuploidy to the next transposon in the genome. Given that all of the blastospore isolates sequenced amplified this region, this region seems to have a high target rate and therefore perturbations should be able to be readily identifiable.

Thank you for raising this important point. We are indeed interested in deleting transposons identified in this study to evaluate their roles in segmental aneuploidy formation. However, transposon movements are very dynamic, and several elements are known to occur in multiple copies across the *Cryptococcus* genome (PMID: 32303657). It will be technically challenging in targeting all copies of these transposons and this topic warrants thorough detailed follow up studies that are beyond the scope of this report.

7. Because the NURAT reporter system requires homologous recombination events that occur during mitosis, it is unclear how this reporter can be used to disentangle the impact of alterations in response to both environment (i.e. media, temperature) and mutation (i.e. cell cycle mutants) on homologous recombination vs. ploidy because the function of the NURAT system is inherently dependent upon both processes to function. Thus, a circular argument is created where the system must first be functioning normally in order to determine dysfunctions.

We appreciate this comment and acknowledge that defects in homologous recombination will limit the use of *NURAT* reporter in detecting ploidy transitions. However, with compromised homologous recombination function, *PCL9* and *CKS1* deletion mutants were able to generate colonies with both the *NURAT* and recombined *NAT* cassettes, suggesting minimal homologous recombination function may support *NURAT*’s application. We revised the text from line 668 to line 671 in discussion to reflect this limitation. We also note that similar types of ploidy sensing reporters were previously developed in *S. cerevisiae* and have been quite effectively applied to study a variety of biologically important processes including ploidy and chromosome segregation and mis-segregation.

8. XL280 is a spontaneous mutant of the wildtype C. deneoformans strain B3501 that has enhanced unisexual mating. Did the authors explore expression of the 6 cell cycle genes in the wildtype B3501 to determine whether the enhanced unisexual mating of XL280 is due to altered expression of one or more of these genes?

Thank you for this great suggestion, we agree these genes may behave differently in B3501 and contribute to the low unisexual mating potential. With all due respect to the reviewer, XL280 is NOT a spontaneous mutant of the wild type strain B-3501. XL280 is an F1 progeny derived from a cross between B-3501 and B-3502, which are themselves the F1 progeny of the environmental isolate NIH433 and the clinical isolate NIH12. Previous studies have shown that pheromone response pathway upregulation plays crucial role in driving enhanced unisexual reproduction potential (PMID: 22737071, PMID: 31536509). The differentially expressed cell cycle regulating genes identified in this study exhibited dynamic expression patterns during the early course of mating induction on V8 (Figure S1) and here we also showed that the ploidy transition during unisexual reproduction is regulated via coordination of these and potentially a broader set of cell cycle regulating genes. It will likely require a comprehensive transcriptomic profiling comparison between strains with high and low unisexual reproduction potential to reveal the intricate regulatory network of cell cycle regulating genes in promoting unisexual reproduction, which we are interested in for follow-up studies.

Minor Concerns:

9. Figure 1A – The breakpoint on the graph is not optimal as it is at a critical point for viewing some of the data. Also, some of the error bars appear to be quite large while others are small. Use of dots to represent spread in the replicates would be more transparent and allow better interpretation of the outliers and replicates that could be influencing the data interpretation and statistical analysis.

We changed the breakpoint to 0.8 and displayed all three data points on the bar graph. All data points are now visible on the revised bar graph.

10. Figure S4 – It is impossible to clearly visualize the 1C and 2C peaks that correspond to each of the individual samples when all the samples are presented on the same plot. Individual plots need to be shown as well as the overlay plot so that the proportion of cells in each individual sample 1C and 2C peak can be easily determined, and then the overlay can be used compare the samples to each other.

To indicate 1C and 2C peaks for each condition for each sample, we generated Table S4 to provide the percentile population under the 1C peak representing G1/S phase and the 2C peak representing G2/M phase. We also used color coding to show the 1C/2C population percentile changes across different conditions for the same sample.

11. Figure 2 – A diagram depicting the difference between blastospores and spores collected from the basidium would be useful. Germination rate can easily be added to Figure 2 and Table 2 can then be moved to the Supplemental Data as most of the information is redundant with the figure.

We added a diagram depicting basidiospores and blastospores in Figure 2. We also added the germination rate in Figure 2 and moved Table 1 to Table S2.

12. Lines 290-91 – Clarify the hypothesis of the experiment and how the results justify the conclusion statement (i.e. because stable diploid colonies were observed Pcl6 is not required for diploid maintenance)

We rewrote the sentence to clarify the conclusion. It now reads: “The observation of stable mitotically passaged diploid cells suggested that *PCL6* is not required for diploid maintenance during mitotic growth”.

13. Lines 301-302 – Include a reference for UAU1

We added the reference for *UAU1*.

14. Figure 4C – Fluconazole resistance in C. deneoformans (or any of the other Cryptococcus species) is not defined. In addition, the most appropriate method for determining changes in susceptibility to fluconazole would be a broth dilution assay, not a spot assay, and determining the IC50 and IC90 for the strains.

We agree the most appropriate method for determining MIC is through either a broth dilution assay or E-test strip assay. However, fluconazole MIC and resistance have been well documented in *Cryptococcus* species, and in particular aneuploidy of several chromosomes, including chromosome 1, can confer fluconazole resistance (Kwon-Chung KJ, Chang YC (2012) Aneuploidy and Drug Resistance in Pathogenic Fungi. PLoS Pathog 8(11): e1003022). Spot dilution assays are also a reliable method in determining antifungal drug sensitivity.

15. Lines 421-424 - …some…all… except… This is a lot of caveats for a single sentence. Recommend simplifying the sentence to read: ….T1 or T3 transposon movements were detected in sequences flanking some of the NURAT/NAT progeny, suggesting that transposable elements may contribute to formation of some of the segmental aneuploidies.

Thank you for this suggestion. We revised the text as recommended.

16. Figure 5 and Lines 478-87 – It is unclear how much the average ploidy calculation is being driven by the outliers with high ploidy. An outlier analysis should be performed and/or analyses thaking these outliers into account either statistically or via comparison/contrast

Thank you for this suggestion. We removed the outliers with ploidies above 1.6 and replotted the data in Figure S11. Statistical analyses supported that deletion of *PCL9* and perturbation of *CLB3* significantly impacted average ploidy among selected colonies.
